# Discovery of Drug Synergies in Gastric Cancer Cells Predicted by Logical Modeling

**DOI:** 10.1371/journal.pcbi.1004426

**Published:** 2015-08-28

**Authors:** Åsmund Flobak, Anaïs Baudot, Elisabeth Remy, Liv Thommesen, Denis Thieffry, Martin Kuiper, Astrid Lægreid

**Affiliations:** 1 Department of Cancer Research and Molecular Medicine, Norwegian University of Science and Technology (NTNU), Trondheim, Norway; 2 Aix Marseille Université, CNRS, Centrale Marseille, I2M, UMR 7373, Marseille, France; 3 Faculty of Technology, Sør-Trøndelag University College, Trondheim, Norway; 4 Institut de Biologie de l’Ecole Normale Supérieure (IBENS), Paris, France; 5 CNRS UMR 8197, Paris, France; 6 INSERM U1024, Paris, France; 7 Department of Biology, Norwegian University of Science and Technology (NTNU), Trondheim, Norway; Swiss Institute of Bioinformatics, UNITED STATES

## Abstract

Discovery of efficient anti-cancer drug combinations is a major challenge, since experimental testing of all possible combinations is clearly impossible. Recent efforts to computationally predict drug combination responses retain this experimental search space, as model definitions typically rely on extensive drug perturbation data. We developed a dynamical model representing a cell fate decision network in the AGS gastric cancer cell line, relying on background knowledge extracted from literature and databases. We defined a set of logical equations recapitulating AGS data observed in cells in their baseline proliferative state. Using the modeling software GINsim, model reduction and simulation compression techniques were applied to cope with the vast state space of large logical models and enable simulations of pairwise applications of specific signaling inhibitory chemical substances. Our simulations predicted synergistic growth inhibitory action of five combinations from a total of 21 possible pairs. Four of the predicted synergies were confirmed in AGS cell growth real-time assays, including known effects of combined MEK-AKT or MEK-PI3K inhibitions, along with novel synergistic effects of combined TAK1-AKT or TAK1-PI3K inhibitions. Our strategy reduces the dependence on a priori drug perturbation experimentation for well-characterized signaling networks, by demonstrating that a model predictive of combinatorial drug effects can be inferred from background knowledge on unperturbed and proliferating cancer cells. Our modeling approach can thus contribute to preclinical discovery of efficient anticancer drug combinations, and thereby to development of strategies to tailor treatment to individual cancer patients.

## Author Summary

Fighting cancer with combinations of drugs increases success of treatment. However, due to the large number of drugs and tumor variants, it remains a tremendous challenge to identify efficient combinations. To illustrate this, a set of 150 drugs corresponds to more than 10.000 possible pairwise drug combinations. Experimental testing of all possibilities is clearly impossible. We have developed a computational model that allows us to identify presumably effective combinations, and that simultaneously suggests combinations likely to be without effect. The model is based on specific cancer cell biomarkers obtained from unperturbed cancerous cells, and is then used to perform extensive automated logical reasoning. Laboratory testing of drug response predictions confirmed results for 20 of 21 drug combinations, including four of five drug pairs predicted to synergistically inhibit growth. Our approach is relevant to preclinical discovery of efficient anticancer drug combinations, and thus for the development of strategies to tailor treatment to individual cancer patients.

## Introduction

It has long been envisaged that future anticancer treatment will adopt combinatorial approaches, in which several specific anti-cancer drugs together target multiple robustness features or weaknesses of a specific tumor [1–3]. The effectiveness of combinatorial anti-cancer treatments can be further maximized by exploiting synergistic drug actions, meaning that different drugs administered together exhibit a potentiated effect compared to the individual drugs. Drug synergy is attractive because it allows for a significant reduction in the dosage of the individual drugs, while retaining the desired effect. Synergies therefore hold the potential to increase treatment efficacy without pushing single drug doses to levels where they lead to adverse reactions. Hence, synergies identified in preclinical studies represent interesting candidates for further characterization in cancer models and clinical trials.

Current efforts to identify beneficial combinatorial anti-cancer therapies typically rely on large-scale experimental perturbation data, either for deciding on specific patient treatment [4], or for pre-clinical pipelines to suggest new drug combinations [5–8]. This work, however, faces challenges posed by the large search space that needs to be supported by experimental data, making systematic searches for efficient combinations challenging. Moreover, the number of conditions for testing dramatically increases when considering higher-order combinations, multiple drug dosages, temporal optimization of drug administration, and diversity of cancer cell types and patients. Thus, workarounds must be sought to reduce the experimental search space of drug combinations and their application modes in order to obtain a qualified repertoire of combination therapies for clinical trials, and ultimately to support delivery of personalized treatment.

Computational models are increasingly used to predict drug effects [6,9], with the aim to rationalize and economize the experimental bottleneck. In order to enable substantial reduction of the number of relevant conditions that need to be tested, such models would ideally be constructed without the need for massive experimental drug perturbation data. Approaches where the formulation of predictive models can be based on molecular data from unperturbed cancer cells are therefore attractive.

We decided to focus on Boolean and multilevel logical models, as they enable a relatively straightforward formalization of the causalities embedded in molecular networks, such as signal transduction and gene regulatory networks. Moreover, logical model simulations can be used to automate reasoning on network dynamics, even with scarce knowledge of kinetic parameters [10–15], and have been used to describe and predict the behavior of molecular networks affected in human disease [13,14]. Such modeling efforts have contributed to the understanding of mechanisms underlying growth factor induced signaling in cancer cells and the selection of candidate target proteins for novel anti-cancer treatment [16–23]. While previous studies have demonstrated the power of logical models to predict single drug actions, we extend the use of logical modeling to predict effects of combinatorial inhibition of two or more signal transduction components.

We report the construction of a logical model encompassing molecular mechanisms central to controlling cellular growth of the gastric adenocarcinoma cell line AGS. After an initial assembly of a comprehensive signaling and regulatory network from general signal transduction knowledge, the logical rules associated with each of the 75 model components were refined using baseline data obtained from actively growing AGS cells. The resulting logical model was used to assess drug synergy potential among 21 pairwise combinations of seven chemical inhibitors, each targeting a specific signaling component. Model simulations suggested five combinations of inhibitors to be synergistic, four of which could subsequently be confirmed in cell growth experiments. Importantly, none of the combinations predicted by the model to be non-synergistic displayed synergistic growth inhibitory effects in our cellular assays, i.e. no false negatives were observed. Our results demonstrate that our logical model, constructed without the use of initial large-scale inhibitor perturbation data, recapitulates key molecular regulatory mechanisms underlying growth of AGS cells in a manner that allows successful prediction of the synergistic effect of inhibitor combinations in experimental cell cultures. Guided by the model, we identified two established synergistic drug interactions and discovered two synergies not previously reported.

## Results

### Overall strategy for the prediction and validation of drug synergies

**Fig 1:**
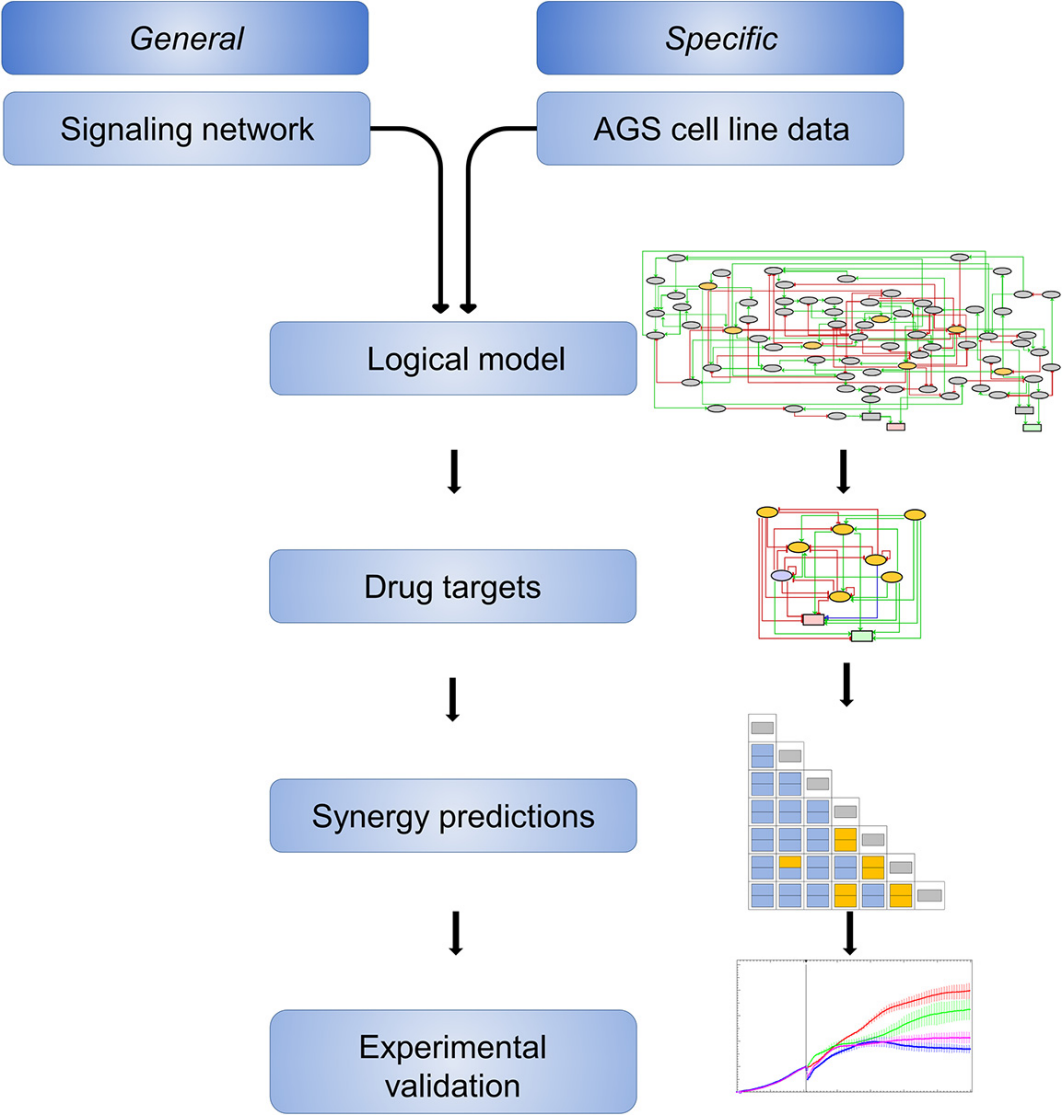
Workflow of model construction and synergy prediction followed by experimental validation. We started with a signaling network built from general database and literature knowledge (upper left), which was refined with published experimental data on protein activities in AGS cells (upper right) to generate the logical model. Next, we generated a formally reduced version of the logical model, focusing on the drug target nodes and valid for systematic simulations of combinatorial inhibitions. Predicted synergies were challenged with observations from AGS cell growth experiments. Cartoons on the right refer to each of the Figs 2–5 further down.

In order to discover combinatorial drug treatments synergistically exerting inhibition of cancer cell growth, we developed a workflow combining computational and experimental analyses to predict and validate drug synergies (Fig 1).

Our modeling procedure integrates *a priori* biological knowledge on intracellular signaling pathways with baseline data from AGS gastric adenocarcinoma cells. The design principles of our analysis are guided by the premise that growth of cancerous cells is largely driven by mechanisms which enable these cells to exploit a wide range of growth promoting signals from the environment. This aspect of intrinsic, sustained multifactor-driven cancer proliferation [1] is accommodated by constructing the regulatory network as a self-contained model: we include only nodes that are regulated by other nodes in the model. The chosen design avoids the need to model effects of specific growth factor receptors, considering instead the integrated responses from a multitude of growth promoting stimuli, as observed when assessing the activity of signaling entities (proteins and genes) included in the model. It follows from this that the *de facto* growth promoting configuration of such a self-contained model can be established by observing baseline biomarkers measured in the cancer cells.

After a model reduction step, where nodes and logics pertaining to drug targets and phenotypic outputs are retained, the model is used for exhaustive simulations of the effect of pairwise node inhibitions using seven known chemical inhibitors. Finally, the growth inhibitory effects of these drug combinations on AGS cells are tested experimentally.

### Logical modeling of gastric adenocarcinoma cell fate decisions

#### Construction of a regulatory graph encompassing key signaling pathways

**Fig 2:**
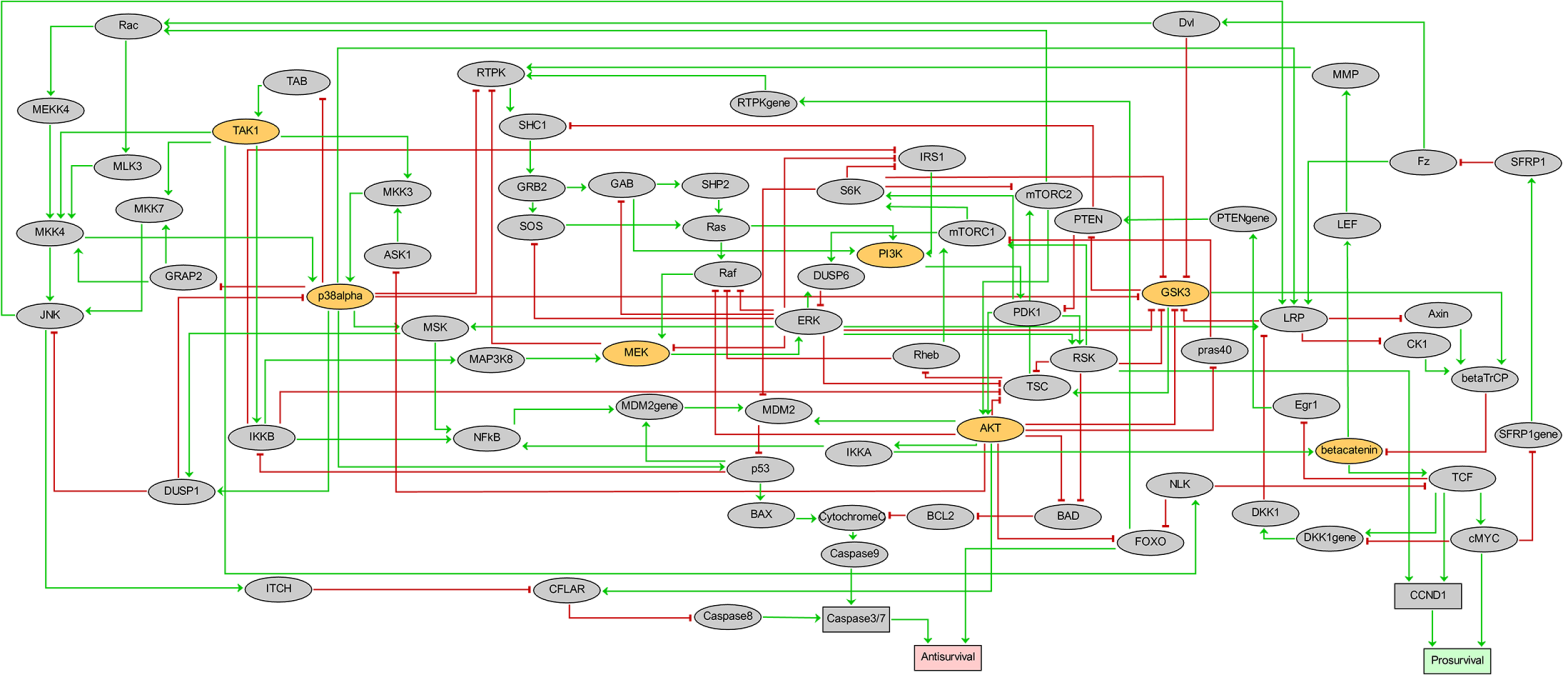
Prior knowledge network representing the cell fate decision network governing growth of AGS gastric adenocarcinoma cells. The network receives no external input but encompasses two outputs *Antisurvival* and *Prosurvival* (phenotypic readouts, colored in red for Antisurvival and green for Prosurvival). Activating regulations are denoted by green arrows, while red T arrows denote inhibition. Signaling component nodes (proteins, protein complexes or genes) associated with Boolean variables (taking the values 0, 1) are represented by ellipses, while rectangles depict nodes encoded with multilevel variables. Yellow nodes represent drug targets and are subjected to inhibitory perturbations during simulations.

AGS cells harbor mutations in numerous genes encoding key signaling components known to be deregulated in gastric adenocarcinoma, for instance components of MAPK, PI3K, Wnt/β-catenin and NF-κB pathways [24,25]. Based on knowledge gathered from databases and scientific publications, we have integrated information about the MAPK pathways (JNK, p38 MAPK and ERK), the PI3K/AKT/mTOR pathways, the Wnt/β-catenin pathway, and the NF-κB pathway, as well as crosstalk between these pathways (see Fig 2, Materials and Methods, and S1 Text). The resulting network comprises 75 signaling and regulatory components (proteins, protein complexes and genes) and 149 directed interactions. Two readout nodes (outputs), named *Prosurvival* and *Antisurvival*, are included to represent cell fate phenotypes. The regulatory graph with annotations is available in SBML format (see S1 Dataset and S1 Table).

#### Construction of a logical model

The regulatory network was converted into a logical model, where the local activity state of each component (node) was represented by a Boolean variable (taking the values 0 or 1). A few nodes were associated with multileveled variables: the two output nodes, *Prosurvival* and *Antisurvival*, each taking four values (0, 1, 2, 3), and their immediate upstream nodes, Caspase 3/7 and CCND1, each taking three values (0, 1, 2). These multilevel variable nodes are only used for nodes governing the outputs of the model, and enabled us to model graded growth promoting/inhibitory effects (see Materials and Methods and S1 Text). A logical formula was associated with each component, defining how its activity level is controlled by those of its regulators. Our default approach was to combine all activating regulators of a target with the Boolean operator *OR*, and inhibitory regulators of a target with the operator *AND NOT* (as in [21]). This implies that any activator can fully activate the target node in the absence of inhibitory activity. Furthermore, the action of any inhibitory regulator can fully inhibit the target, even in the presence of activating input from one or more activators. On the basis of biological knowledge and literature reports, more specific rules were defined for some components of the model (see S1 Text). For the β-catenin pathway in particular, we refined logical rules of nodes representing activity of β-TrCP (the β-catenin destruction complex), TCF (a target of β-catenin activity), and the node representing activity of β-catenin itself.

At any time, the global state of the system is represented by a discrete vector containing the Boolean or multilevel activity values for all network components [26]. As all node states are iteratively updated in simulations the model converges to its attractors, represented by single global fixed states in simple attractors, or sets of states repeatedly traversed in complex attractors. Based on the regulatory graph and logical rules defined above, we used a powerful algorithm implemented in GINsim to compute all stable states of the model.

To calibrate the model with respect to actively growing AGS cells, we compared node state predictions against AGS baseline biomarker observations reported in the literature. We reviewed 72 scientific publications and found 219 experiments with proliferating AGS cells providing information on the activity of proteins represented in our model (see S1 Text and S2 Table). We chose a subset of 21 proteins for which the activity data was supported by several independent but consistent reports. Using these experimental observations as guidelines for “gold standard” protein activities in actively growing AGS cells, we compared the state of each of them with their level in the computed attractor of the model. To obtain a single stable state containing activity levels of all model components, the logical rules of components of the ERK pathway (SHC1, SOS, Raf, MEK and ERK) were defined to reflect the observation that ERK is active in proliferating AGS cells (see S1 Text and S2 Table). After these modifications, the model proved to be optimized: the observed attractor of the unperturbed model was a stable state thoroughly corroborated by experimental observations in unperturbed growing AGS cells, as the values of all the 21 nodes that we were able to check match reported protein activities (see S1 Text, S3 and S4 Tables). In addition, the value of the readout nodes *Prosurvival* was at its maximum, and Antisurvival at its minimum, representing strong proliferation (*Prosurvival* = 3, and *Antisurvival* = 0). This model stable state is thus consistent with published knowledge about molecular states in actively growing AGS cells. This model also complied with results from published perturbation experiments of AGS cells (see S1 Text and S6 Table). The resulting logical model, encoded with the software GINsim v2.9, is shown in Fig 2. The corresponding GINsim file is provided as S2 Dataset.

### *In silico* simulations predict five inhibitor synergies

In order to assess combinations of inhibitions for synergy, we focused on the systematic inhibition of seven model nodes and their 21 pairwise combinations. These seven nodes (labelled with thick borders in Fig 2) were chosen because potent and specific chemical inhibitors were available for targeting the corresponding protein kinases in biological experiments (Table 1).

**Table 1:** Chemical inhibitors and their corresponding protein kinase targets.

*Chemical inhibitor*	*Target name*	*Target HGNC symbol*	*GI50* *
(5Z)-7-oxozeaenol	TAK1	MAP3K7	0.5 μM
AKTi-1,2 (AKT inhibitor VIII)	AKT1/2	AKT1, AKT2	10 μM
BIRB0796	p38 MAPK	MAPK14	N/A (5 μM used) **
CT99021	GSK3	GSK3A, GSK3B	N/A (5 μM used) **
PD0325901	MEK	MAP2K1, MAP2K2	35 nM
PI103	PI3K	PIK3CA	0.7 μM
PKF118-310	β-catenin	CTNNB1	150 nM

* Experimentally determined concentration that inhibits AGS cell growth by 50% (GI50).

** For the two inhibitors BIRB0796 and CT99021 no GI50 could be obtained, and 5 μM was chosen as a concentration that is expected affect their target in our experimental setup, based on observed effects in similar cell systems [27]. See S1 Text for further documentation of inhibitor properties.

**Fig 3:**
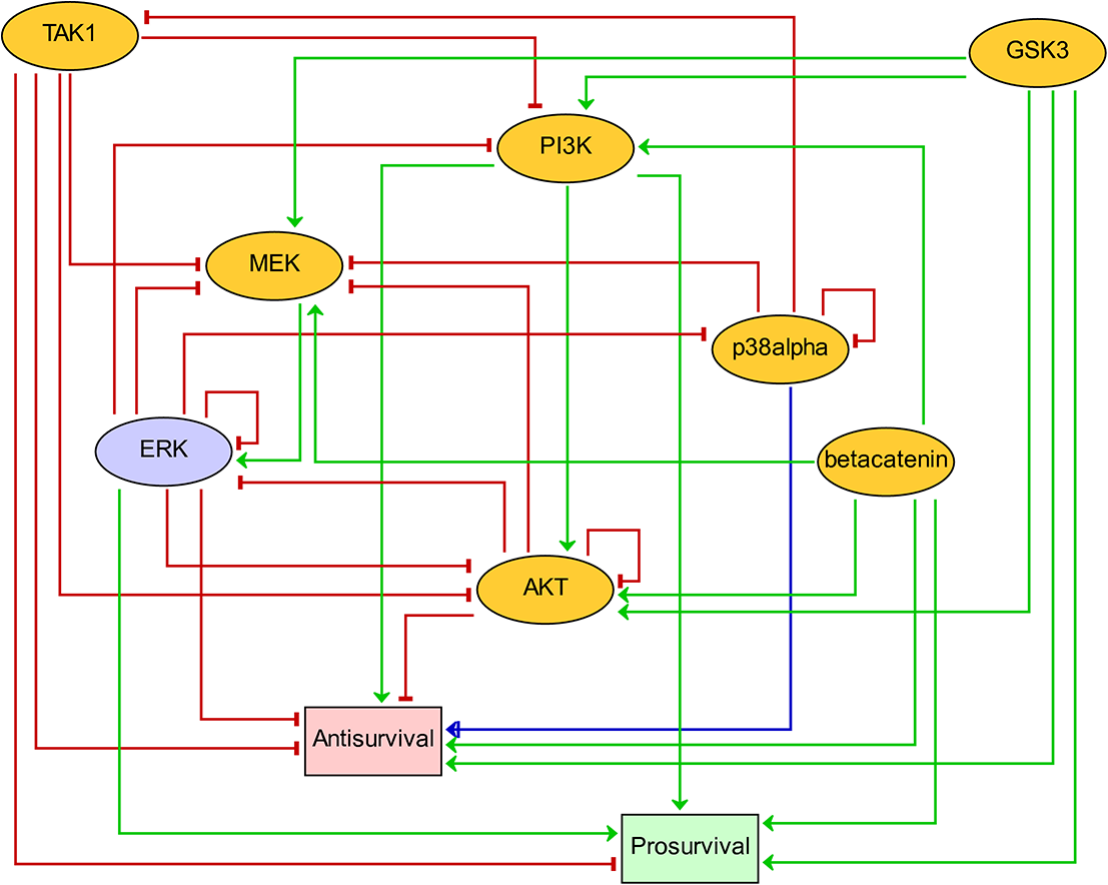
Reduced logical model obtained by semi-automated reduction of the comprehensive logical model shown in Fig 2. The reduced model encompasses all seven drug targets (yellow) and the two phenotypic outputs (red for Antisurvival and green for Prosurvival). In addition the ERK node (blue) had to be preserved to maintain dynamical consistency with the large model. Activating regulations are denoted by green arrows, while red T arrows denote inhibition. The blue arc with both arrow and T head (p38alpha to Antisurvival) indicates a dual regulation, i.e. activating and inhibiting, depending on context. In some contexts p38alpha inhibition will increase Antisurvival, while in others p38alpha inhibition will decrease Antisurvival (see S1 Text, S7 and S8 Tables). Note that after model reduction two members of the Wnt/β-catenin pathway, β-catenin and GSK3, became non-regulated and fixed at either the on-state (β-catenin) or off-state (GSK3).

Using an asynchronous updating policy (see Materials and Methods), we simulated the effect of chemical inhibitions by forcing the state of specifically targeted model nodes to be 0 (inactive), and then computing the resulting attractor. Each inhibition of single nodes or pairs of nodes led to a unique attractor. In a few cases the system reached a complex attractor, in which a subset of states is traversed repeatedly (see Materials and Methods and S1 Text). The computation of potential complex attractors is challenging because of the combinatorial explosion of states for large logical models. To cope with this problem, we used a model reduction method to obtain a compressed model preserving the selected drug targets, and compacted the state transition graphs in a hierarchical manner (see Materials and Methods and [14]). The reduced logical model (see Fig 3 and S3 Dataset) was obtained by iteratively removing components not targeted by drugs, and was sufficiently small to allow exhaustive asynchronous simulations and thorough characterization of both stable states and complex attractors, thereby enabling the analysis of all single and pairs of inhibitions.

To ease interpretation, we defined the overall response *growth*, by subtracting the value of *Antisurvival* from the value of *Prosurvival* readout nodes (each multi-valued with state ranging from 0 to 3), with a value range from -3 to +3. If the attractor contained a unique stable state, the computation of *growth* was straightforward. In the case of complex attractors we used the mean values of the difference *Prosurvival*–*Antisurvival* over all states belonging to the attractor. We inferred synergy whenever the combination of two inhibitors produced a value for *growth* lower than the smallest value of the inhibitors individually:

growth (perturbation1 & perturbation2) < Min (growth (perturbation1), growth (perturbation2)),

where *perturbationN* is the perturbation of component *N*.

For example, *growth (perturbationMEK & perturbationAKT)* = 0.5; which is a value lower than observed with perturbations of either MEK or AKT: *growth(perturbationMEK)* = 1.5; *growth (perturbationAKT)* = 2.

**Fig 4:**
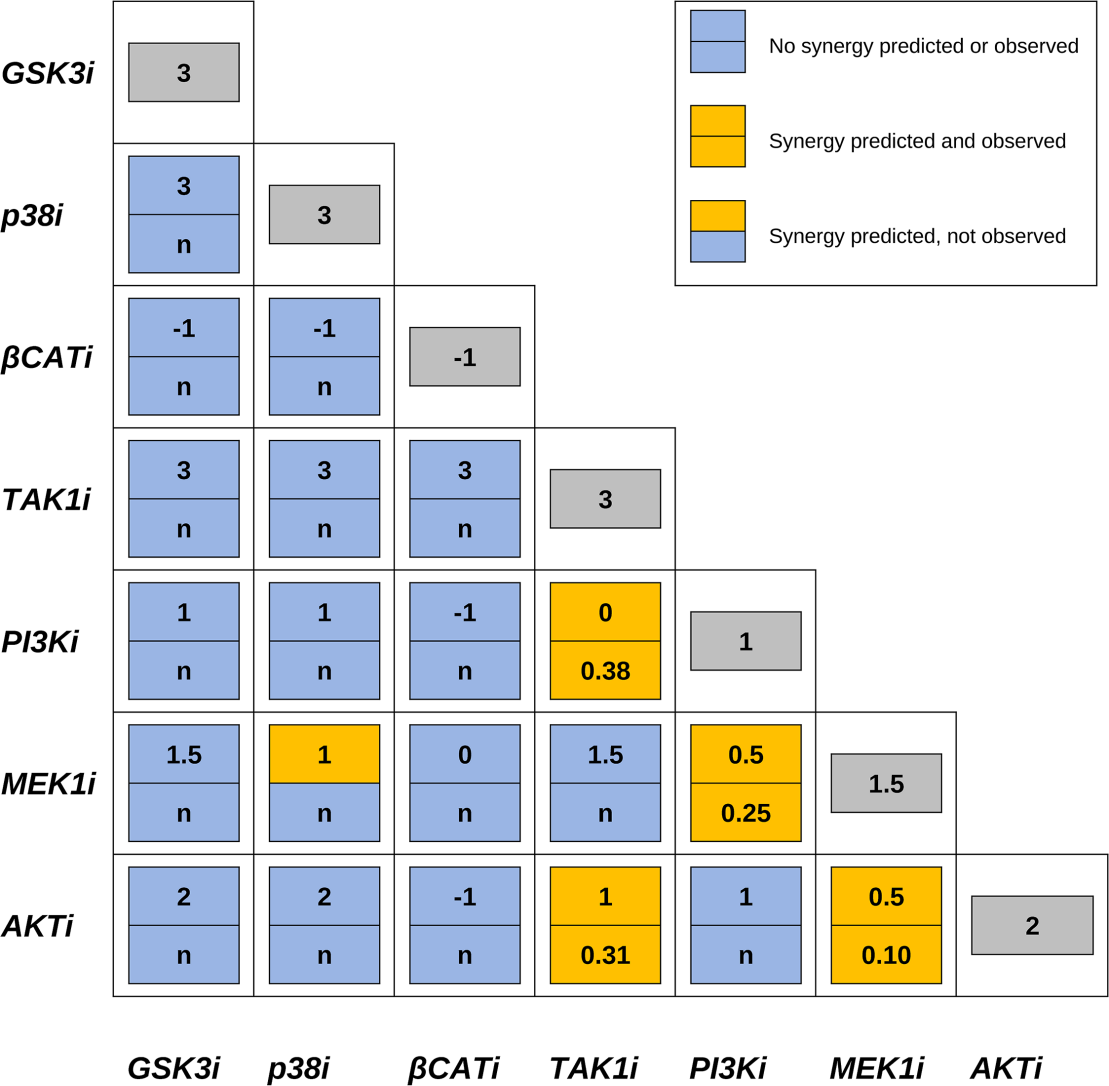
Effects of combined inhibitors on cell growth. Synergistic (yellow) and non-synergistic (blue) combinations are shown both as predicted by model simulations (upper panel of boxes, value of model parameter “growth”) and as verified by cell growth experiments (lower panel of boxes; combinatorial indexes (synergy indicated by CI < 1) or “n” when non-synergy was observed).Synergy was proposed whenever the predicted growth for a combination of inhibitors was lower than the modeled effect of single drug perturbations, shown in the outer diagonal (grey, value of model parameter “growth”).

The simulations predicted five synergistic combinations (<25% of the 21 possible pairs). Three of these combinations involve MEK, together with PI3K, AKT or p38. The two remaining synergies involve TAK1 with either PI3K or AKT (Fig 4).

### Experimental validation of model predictions

To assess the validity of our model predictions, a real-time cell assay was used to test chemical inhibitors of the seven proteins (Table 1) for their ability to limit AGS cell growth in single and combinatorial formulations.

The effect of chemical inhibitors was analyzed using a strategy based on Loewe’s definition of synergy [28], which states that a synergistic interaction performs better than the expected additive effect observed when an inhibitor is combined with itself in a ‘zero-interaction’ experiment. To quantify synergistic interactions, a combinatorial index (CI) was calculated [29], based on growth measured 48 hours after adding inhibitors. CI values range from zero to infinity, and values below 1 indicate synergistic interactions.

**Fig 5:**
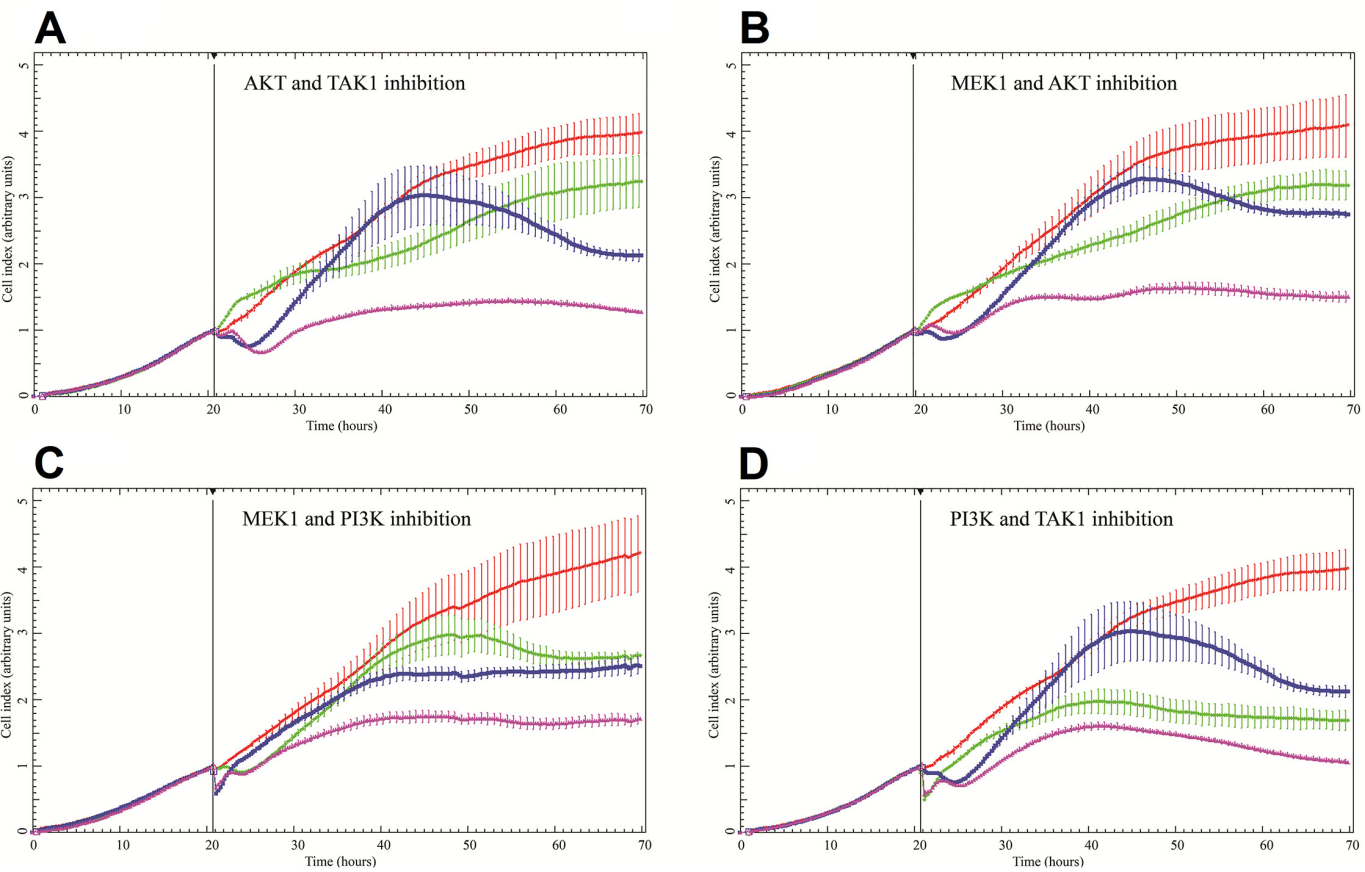
Experimentally confirmed synergies, where the effect of combining two inhibitors at half GI50 concentrations (violet) outperforms each of the single inhibitor at the full GI50 concentration. A) AKT inhibitor (green) and TAK1 inhibitor (blue). B) MEK inhibitor (blue) and AKT inhibitor (green). C) MEK inhibitor (green) and PI3K inhibitor (blue). D) PI3K inhibitor (green) and TAK1 inhibitor (blue). Cells growing in the absence of inhibitors are shown in red. One standard deviation is indicated by error bars. Inhibitors (and concentrations) used: MEK inhibitor PD0325901 (35 nM), TAK1 inhibitor (5Z)-7-oxozeaenol (0.5 μM), PI3K inhibitor PI103 (0.7 μM) and AKT inhibitor AKTi-1,2 (10 μM). See Materials and Methods and S1 Text for all growth curves of combinations of inhibitors, and dose-response curves of individual inhibitors.

Four of the five synergies predicted by our logical model were confirmed experimentally, with CI values well below 0.5, which indicates strong synergy. Indeed, a profound effect on AGS cell growth was found when MEK or TAK1 inhibitors were combined with PI3K or AKT inhibitors. The corresponding growth curves (Fig 5) indicate that cell growth in the presence of two inhibitors combined, each at half their GI50 concentrations (purple curves) is markedly lower than growth in the presence of either inhibitor alone at its full GI50 concentration (green and blue curves). In contrast, the combination of MEK and p38 inhibition could not be confirmed in the cell growth experiments. Importantly, we observed no false negative predictions, meaning that the remaining inhibitor combinations, predicted to lack synergetic effects, indeed failed to display synergy in our cellular assays. Taken together, our model simulations proved to be highly accurate, correctly predicting the effects of 20 of the 21 combinations.

Synergies of PI3K-MEK or AKT-MEK inhibitions have already been observed in a variety of tumor cells [6,30–34], thus providing further confidence to the synergies of TAK1-PI3K and TAK1-AKT inhibitions. Hence, these novel combinatorial inhibitions are promising candidates readily amenable to experimental testing in a range of cancer cell types.

### Model suggests a key role for FOXO in growth inhibition synergy

Understanding the signaling mechanisms underlying synergistic inhibitions is of high interest because it can contribute to the identification of biomarkers informative of treatment response, which may serve as guides to select from an arsenal of established drug synergies the right treatment for the individual patient.

Examination of simulated perturbation effects with our AGS logical model revealed that FOXO, representing pro-apoptotic transcription factors inactivated by phosphorylation [35], was synergistically activated by combined MEK and PI3K or MEK and AKT inhibition (see S1 Text). Interestingly, single inhibitory perturbation of MEK, PI3K or AKT did not change FOXO activity (see S1 Text for more details). These observations match experimental findings in human umbilical vein endothelial cells (HUVEC), where inhibitors targeting MEK and AKT are reported to synergistically activate FOXO [36], which suggests that our model simulations can provide a basis for biologically relevant hypotheses on molecular effects downstream of specific inhibitors.

To further investigate the mechanisms involving FOXO we simulated the inhibitor perturbations in a FOXO knock-out model, and found that combined inhibition of MEK and PI3K displayed no enhanced growth inhibitory effect compared to their corresponding single inhibition. This indicates that the MEK-PI3K synergy indeed depends on FOXO. For the combined MEK and AKT inhibition, the FOXO knock-out model simulations showed only a minor reduction of the synergistic effect of combined MEK and AKT inhibition. The synergy between MEK and AKT inhibitors thus appears to be less dependent on FOXO. Taken together, these simulation results suggest potentially interesting differences between pro-apoptotic signaling events when comparing MEK-AKT inhibition versus MEK-PI3K inhibition.

The mechanistic basis of the synergies observed when inhibiting TAK1-AKT, or TAK1-PI3K, is unknown. Interestingly, AGS model simulations show that FOXO is activated when TAK1 is inhibited in combination with either PI3K or AKT, but not by single inhibitions. Activation of FOXO is thus a potential mediator also for the synergies involving TAK1. In support of this, simulations showed that both TAK1-PI3K and TAK1-AKT synergies were abolished when FOXO is knocked-out, similarly to the finding of MEK-PI3K inhibition (See S1 Text). Potentially, ERK could be involved in signaling downstream of TAK1. In that case the MAP kinase cascade could represent a common mechanism implicated in the synergies involving MEK-PI3K and MEK-AKT, and those involving TAK1-PI3K and TAK1-AKT. However, our AGS model simulations predict that ERK is still active after combined inhibition of TAK1 and PI3K or of TAK1 and AKT. This may indicate that MEK/ERK is not involved in the downstream effects of the inhibitory perturbations involving TAK1. Another kinase could potentially function as a point of crosstalk for TAK1 and PI3K/AKT signaling. In this respect, NLK (Nemo-like kinase) is an interesting candidate as it is known to act downstream of TAK1 [37], mediating inhibitory phosphorylation of FOXO [38].

The model-based suggestion that FOXO activation may be important for synergistic growth inhibition does find experimental support in numerous accounts of FOXO proteins acting as mediators of cytotoxic chemotherapeutic drugs [39]. This suggests that the dynamical behavior of our logical model recapitulates generic properties that may be relevant for a range of different tumor types.

## Discussion

The development of novel anti-cancer medication predominantly focuses on drugs directed against specific molecular targets. However, clinical applications have often been disappointing, resulting only in transient responses followed by drug resistance which hinders therapy benefits. This has led to the consideration of therapies based on combinations of drugs targeting different signaling pathways or cellular processes, with the aim to restrain the evolution of drug resistance and at the same time allow for a reduction in drug dosage, to lower drug-induced toxic effects [2,3,40,41].

These strong incentives for combinatorial drug treatment are challenged by the numerous combinations to consider and by the fact that the efficacy of a given drug combination is dependent on the nature of the specific tumor. Thus, to discover apt drug combinations at a pace compatible with the vast search space posed by the many drugs and diverse cancer cell spectrum, it is mandatory to develop efficient strategies to predict beneficial combinatorial treatment for individual cancers.

Current efforts to come to a rational choice of drug combination therapy by using primary tumor cell cultures and xenograft studies are confronted by high costs and a variable rate of success in tumor cell growth inhibition, and struggle to obtain highly accurate predictions within the timeframe limited by disease progression [4,42–44]. While cancer cell line cultures rarely allow for discoveries that can be directly transferred to a clinical setting, they do allow for experimental investigation of mechanisms underlying biological diversity and robustness and can thus be used to explore strategies to identify potentially effective drug combination therapies. They can therefore contribute to establish a large arsenal of advantageous drug combinations accompanied by prognostic tools enabling the choice of the right combination for the individual tumor. However, even in these cellular models, it is not feasible to test all potential drug combinations and application modes for a sufficient spectrum of cancer cell types. In this context, computational modeling can be of great help to reduce the experimental search space.

We have demonstrated how a logical model built from known signal transduction network information can be tailored to a specific cancer cell system using baseline data, so that it can be used to predict synergistic and non-synergistic combinatorial growth-impeding treatments. Four of the five predicted synergistic combinations were confirmed experimentally with no false negative predictions. With such a success rate, it would have been sufficient to test only a quarter of the 21 possible drug combinations investigated and still not miss any synergistic pair. Our results are encouraging in light of the success rate reported from the recent DREAM challenge [7], where the best-performing method of synergy prediction would have allowed halving the size of screening experiments. However, there are important differences between our study design and that of the DREAM challenge: DREAM analyzed transcriptome changes following broad-acting chemotherapeutic drug treatments, while we investigated the action of inhibitors with specific targets, relying only on information from the unperturbed system.

Contrary to network-based strategies, which commonly use correlation analysis of large-scale datasets from different disease phenotypes [45,46], or large-scale cell culture drug perturbation data to train models for drug response predictions [6,7,9,47,48], our modeling-based strategy exploits mechanistic molecular pathway knowledge, available in databases, along with baseline data from the unperturbed cancer cells of the chosen experimental system. This means that our approach allows for the selection of interesting candidates for efficient drug combinations before performing actual drug perturbation experiments. To our knowledge this has not been successfully demonstrated before.

The majority of regulatory network modeling approaches focus on signaling events driven by specific hormone receptors. This applies to studies investigating logical modeling to understand consequences of interfering with specific growth factor signal transduction responses [16,18,49–51], as well as to quantitative and semi-quantitative modeling approaches used to predict the effect of synergistic signal transduction perturbations [6,31,52]. In contrast, our approach demonstrates that it is possible to effectively use a model representing a cell fate decision network in actively growing cells without considering explicitly any external growth-promoting stimulus (e.g. growth hormone). Indeed, we argue that using the attractor of a self-contained model of a proliferating cell as the reference point for drug synergy analysis provides a good proxy for the state of actively growing cancer cells. Cancer cell growth is considered to be driven by a multitude of growth promoting stimuli. Not only is the potential repertoire of these signals substantial, relatively little detail about their signaling mechanisms is known. We therefore assume that we can summarize their effect by considering this multitude of signals to provide a context promoting robust growth and that we can therefore dismiss any further detail. On this basis we accommodate a sustained multifactor-driven proliferation [1] by employing a self-contained model, where all components included are regulated by other nodes in the model. The configuration of component activities can then be inferred from baseline biomarkers measured in cancerous cells. Together, these model design principles enable us to generate a dynamic model tailored to specific cancer cells, yet not dependent on explicit extracellular input from specific growth promoting agents (*e*.*g*. growth hormones) and without the need for initial large-scale inhibitor perturbation data that would be difficult and costly to obtain.

Our definition of drug synergy in experimental validation is based on Loewe additivity [28], and synergies are quantified with the combinatorial index [29]. Several mathematical frameworks have been proposed to determine drug synergy [53]. We chose the Loewe method as it is extensively used, and it correctly handles the sham zero-interaction experiment where a drug is ‘combined with itself’. Regarding the predictions from the logical model, we identify potential synergies by selecting drug pairs that have a more profound effect on the global output ‘growth’ than either of the single drugs. While the experimental synergy analysis allows quantifying the degree of synergy, the logical model is discrete and cannot provide synergy quantifications. Even though our definitions of synergy in experiments and simulations are not identical, our model-based classification proved to be highly accurate with regard to synergies assessed experimentally. Tentatively, a translation of logical variables into continuous ones could be considered (see for example [54]), to estimate synergies in a manner more analogous to the computation of experimental combinatorial indexes.

The AGS gastric adenocarcinoma cell line was chosen as a model system because its gene expression profile is highly similar to profiles of gastric adenocarcinoma [55,56] (intestinal subtype, Lauren’s histopathological classification). Despite increased understanding of the molecular underpinning of gastric cancer, it remains the second leading cause of cancer death globally and as such an austere reminder of the need for improved treatments [57]. In Western countries, two thirds of gastric cancer cases are discovered at a stage where radical treatment is not feasible, and more than half of the patients who can be radically treated will experience relapse. For patients with advanced gastric cancer, the 5 year survival is less than 10% [58].

The synergy between PI3K and MEK inhibitors in AGS cells is in line with previously published observations [6,30,31,33], and is currently being pursued in clinical trials for advanced solid cancer (including pancreatic, breast, non-small cell lung cancer and colorectal cancer) [59]. Similarly, the synergistic effect of MEK and AKT inhibitors has been previously observed [32,34], and is currently investigated in clinical trials (including multiple myeloma, breast, endometrial, colorectal, non-small cell lung cancer, pancreatic cancer, ovarian cancer) [60]. The novel growth inhibitory synergies between TAK1-PI3K and TAK1-AKT discovered here are interesting candidates for further investigations.

Our knowledge concerning molecular regulatory mechanisms underlying cell fate decision networks is rapidly increasing. This poses both opportunities and challenges to integrate many details into an extensive understanding, enabling global mechanistic reasoning on regulatory networks and construction of comprehensive models that can provide *in silico* predictions of drug effects. Translation of these predictive capabilities to clinical settings is facilitated by the increasing availability of patient omics data, which can provide biomarkers informative of cellular signaling status associated with disease and treatment response. The integration of biomarker information with models of combinatorial drug responses may provide important clues to improve health care for patients who currently lack effective treatment.

## Materials and Methods

### Construction of the signaling network

We used pathway information from databases as a source of signaling components, and included interactions for MAPK pathway (JNK, p38, ERK), the PI3K/AKT/mTOR pathway, the Wnt/β-catenin pathway and the NF-κB pathway. Each protein was annotated with its official gene symbol, Uniprot protein identifier, and mutational status in our AGS gastric adenocarcinoma experimental system. All interactions were substantiated by bibliographical references documenting experimental evidence. The model was composed of 75 signaling components and 149 directed interactions.

### Logical modeling analysis and simulations

#### Logical modeling

To perform simulations, we used the logical formalism initially proposed by René Thomas [61]. This approach starts with the definition of a regulatory graph, wherein each node represents a model component, and each directed arc represents an (activating or inhibitory) interaction between two components.

The activities of all but four components are associated with Boolean variables (variables taking only the values 0 or 1). The corresponding discretization reflects the “threshold effect” of the regulatory interactions between components: a component is considered “active” (Boolean variable equals 1) when its activity level (concentration or catalytic activity) is sufficient to affect the activity of other components in the system. As long as this activity level is not sufficient to exert an effect, the component is considered inactive (and the corresponding Boolean variable equals 0).

The phenotypic output nodes *Prosurvival* and *Antisurvival* were allowed to occupy the four levels from 0, 1, 2 and 3, to capture the relative effects of converging combinations of activators and inhibitors. For example, if Caspase 9 (representing the intrinsic apoptotic pathway) is active, while Caspase 8 (representing the extrinsic apoptotic pathway) and FOXO are inactive, *Antisurvival* takes the value 1. If all three regulators (Caspase 8, Caspase 9, and FOXO) are active, the value of *Antisurvival* will be 3. Furthermore, Caspase3/7 and CCND1, direct regulators of the output nodes *Antisurvival* and *Prosurvival*, respectively, were associated with ternary variables (taking the values 0, 1 or 2). The value of the variable associated with Caspase3/7 is given by the summation of those corresponding to Caspase 8 and Caspase 9. For CCND1 the ternary variable represents the integration of RSK and TCF signaling.

Logical rules define the evolution of the activity level of a component depending on those of its regulators, using formulae with classical Boolean operators *AND*, *OR*, and *NOT*. Our default approach was to combine all activating regulators of a target with the Boolean operator *OR*, and all inhibitory regulators of a target with the operator *AND NOT* (as in [21]). This implies that any activator can fully activate the target node if no inhibitory interactions are active. Conversely, the activity of any inhibitory regulator can fully inhibit the target, even when the target receives input from activators. To have a dynamical behavior consistent with available data, we had to refine the rules determining activity of several components:

1. betaTrCP (representing the activity of the β-catenin destruction complex, which promotes β-catenin ubiquitination and degradation): we used an *AND* operator to represent the fact that all components of the β-catenin destruction complex are simultaneously needed to lead to β-catenin degradation [62].
2. betacatenin: the rule was refined to represent the fact that degradation can be protected by phosphorylation by IKKA [63,64].
3. SHC1, SOS, Raf, MEK and ERK: inhibitory regulators were connected with *OR NOT* instead of the default *AND NOT*, in order to reduce the impact of the negative regulatory circuit present in the SOS/MEK/ERK pathway. With these refined rules, model simulation in the unperturbed case leads to a single stable state with ERK active, which better matches published experimental observations for AGS cells [65,66].
4. TCF: the impact of negative regulation by NLK was reduced so that TCF was active in the unperturbed case, in line with published data [55,66].

A listing of all model components and logical rules is provided in S5 Table, along with a model file to be opened with the software GINSIM v2.9 or newer (model in S2 Dataset).

#### Logical simulations

Using GINsim [67], we can compute the dynamical behavior of our AGS model for any initial state. The state of each model component is then iteratively updated, according to the logical formulae.

The resulting dynamics is represented in terms of a state transition graph (STG). The nodes of the STG denote the states of the system, i.e. discrete vectors encompassing the activity values of all components (Boolean variables, except for the four multi-valued components), while the arcs connect successive states, denoting “state transitions”. Enabled transitions were defined based on an asynchronous updating policy: whenever multiple components are called for a change, all single value changes are considered, leading to the representation of all possible asynchronous trajectories in a single STG.

The asymptotic behavior(s) of the system corresponds to the attractors of the dynamics (terminal strongly connected components in graph theoretical terms). Since our AGS model is finite, its dynamics contains at least one attractor. Two types of attractors may occur: stable states (single state attractors) and complex (cyclic) attractors (sets of states from which the system cannot escape). Stables states can be easily computed using Multi-valued Decision Diagrams [26]. To identify and characterize the complex attractors for such a large network, we have combined a model reduction approach [26] with a method enabling the compaction of state transition graphs into hierarchical transitions graphs [14].

#### Model reduction

The model reduction method consists of iteratively replacing selected components of the network by an updated logical function of their target nodes [26]. Our reduced model was configured to preserve the output nodes, the seven targets of experimental perturbations, and the nodes involved in self-loops (self-loops may be introduced during the reduction process). The stable states are conserved by this reduction. Furthermore, each complex attractor of the original model is matched by at least one complex attractor in the reduced model. However, as model reduction generally results in a simplified STG (elimination of transitions, considered as instantaneous), complex attractors may be split during the reduction process, while attractor reachability might also be affected [26]. Such distortion of the dynamics was assessed by checking the behavior of the original model or by using alternative reductions. For all reduced instances of the model, we found either a single stable state or a single complex attractor.

#### Hierarchical state transition graphs

The analysis of state transition graphs becomes intractable as their size increases. To ease its interpretation and its manipulation, an STG can be compressed into a hierarchical transition graph (HTG), which preserves its main structural properties: as the STG is constructed, its nodes are gathered into groups of states sharing the same set of successors [14]. The resulting HTG displays all reachable attractors, and their basins of attraction.

#### Simulation of model perturbations

Using the logical formalism and GINsim, it is relatively straightforward to encode model modifications that account for different kinds of genetic or pharmacological perturbations: Simulation of drugs blocking the activity of a component, or knock-out of genes encoding specific components, is performed by fixing the activity value of the drug target component to the Boolean value 0.

In short, each perturbation is encoded in terms of a well-defined rewriting of the logical rule(s) of the corresponding component(s). In this respect, GINsim includes an interface enabling the definition and the storage of single perturbations (mutations), as well as various combinations thereof.

### Cells and reagents

AGS (human gastric adenocarcinoma, ATCC, Rockville, MD) were grown in Ham’s F12 medium (Invitrogen, Carlsbad, CA) supplemented with 10% fetal calf serum (FCS; Euroclone, Devon, UK), and 10 U/ml penicillin-streptomycin (Invitrogen). Growth experiments were performed with medium with 5% FCS.

Chemical inhibitors PI-103 (Merck), AKT-i-1,2/AKT inhibitor VIII (Merck), PD0325901 (Sigma-Aldrich), PKF118-310 (Sigma-Aldrich), BIRB 0796 (Axon), and CT99021 (Axon) dissolved in DMSO at stock concentrations of 20 mM, except PI103 which was dissolved in DMSO at a stock concentration of 10 mM.

### Growth measurements

Cell growth was measured without labelling, in real-time, with the xCELLigence RTCA SP (96-well) or xCELLigence RTCA DP (16-well) growth assay (Roche Applied Science). This system utilizes culture plates with gold electrode arrays at the bottom of each well in multi-well E-plates (Roche Applied Science). Real-time measurements of the impedance across the gold arrays were reported in the dimensionless unit of cell index which is taken to correspond to the number of cells. In agreement with manufacturer’s instructions, cells were split 1:1 the day before experiments to ensure that cells were in an exponential growth phase at the time of seeding cells for xCELLigence analysis. First, complete medium was added to wells in 50 μl aliquots to measure background signal, next 100 μl of cell suspension was added, at a seeding density of 5x10^3^ cells per well. The well plate was then put back in the RTCA SP/DP instrument, where cells are allowed to adhere overnight (20 hours). The well plate was then removed from the instrument, and 50 μl aliquots of complete medium with chemical inhibitor of interest were added to each well, to a total volume of 200 μl. Real-time monitoring of cell proliferation was performed for 72 hours, at which time the effect of growth arrest was stable (see S1 Text).

### Experimental assessment of inhibitor synergy

#### Determination of GI50

For each inhibitor a dose-response profile, including the 50% growth inhibitory dose (GI50) was determined (see S1 Text).

In general, our results for AGS cells fit those found by other groups for the same or similar cell lines (see S1 Text).

#### Analysis of combinatorial treatment

We used Loewe’s definition of synergy, which states that a synergistic interaction is one that performs better than the expected additive effect [28]. Taking the GI50 of each compound as the reference concentration in the assessment of synergy, each combination of two inhibitors was tested. For two different drugs at equipotent concentrations, the activity is *additive* if the combined effect of two drugs at half concentration restores the effect of either drug at the full concentration, and *synergistic* if the combined effect outperforms the effect of either drug at the full concentration. All inhibitors were tested in combinations of 0.5x GI50 concentrations, and two two-fold dilutions (0.25x and 0.125x GI50). Single inhibitors were also tested in the same experiment at GI50 concentrations and two two-fold dilutions (0.5x and 0.25x GI50). Whenever the combination of two drugs at 0.5x GI50 was more effective than either of the single drugs at 1xGI50, a synergistic combination was declared.

The combination index (CI) was calculated for each combinatorial experiment according to Chou and Talalay [29], using the CompuSyn software [68]. CI was calculated from growth measured at 48 hours after adding inhibitors, because at this point the growth inhibitory effect had reached a stable level (see S1 Text). The combinatorial index is a mathematical description of synergy, based on a median-effect plot made from specific dose-response data [68]. A CI value of 1 is taken to indicate an additive effect. A CI value over 1 is considered to indicate antagonism, and conversely a CI value below 1 is considered to indicate synergy.

## Supporting Information

S1 Text Supporting information. Details of curation of pathways and conversion to logical model. Analysis of model compliance with literature-derived signal transduction data of AGS cells. Cell growth experiments, with dose-response curves for each of the seven inhibitors, and growth curves of 21 combinations of inhibitors. Description of model analysis of FOXO knockouts and its effect on predicted synergies.
(PDF)

S1 Dataset SBML model. The model includes annotations for each node entity with Uniprot IDs, official gene symbols, gene mutations in AGS, and mRNA in AGS. Node interactions are referenced with publication IDs. The model was created with CellDesigner v4.3 (http://celldesigner.org/).
(XML)

S2 Dataset GINsim model. The model can be opened with the open-source software GINsim (http://ginsim.org/). The model includes annotations for each node with Uniprot IDs, official gene symbols, gene mutations in AGS, and publication IDs for published reports on state of modeled protein in proliferating AGS cells. Node interactions are referenced with publication IDs.
(ZGINML)

S3 Dataset GINsim model. Reduced model file, obtained from the full model in S2 Dataset using built-in model reduction capabilities of GINsim, and with logical rules as shown in S8 Table. The model reduction was configured to preserve nodes targeted by drugs, nodes involved in self-loops, and the two phenotypic output nodes.
(ZGINML)

S1 Table HGNC symbols. Conversion table for model node names and corresponding HGNC symbols.
(XLSX)

S2 Table Literature review of baseline signaling data in AGS cells. References to publications describing 219 observations of steady state signaling in AGS cells.
(XLSX)

S3 Table Comparison of stable state of logical model and literature-derived steady state signaling. (XLSX)

S4 Table Summary of literature-derived steady state signaling observations. (XLSX)

S5 Table List of 77 logical equations encompassed by GINsim model. (XLSX)

S6 Table Literature review of perturbation experiments in AGS cells. References to publications describing 56 observations of signal transduction perturbation responses in AGS cells.
(XLSX)

S7 Table Logical parameters for reduced logical model. (XLSX)

S8 Table Compressed logical rules for reduced logical model. (DOC)
