# Supplementary material for: Discovery of Drug Synergies in Gastric Cancer Cells Predicted by Logical Modeling
Source: PLoS Comput Biol. 2015 Aug 28;11(8):e1004426. doi: 10.1371/journal.pcbi.1004426 (PMC4567168; doi:10.1371/journal.pcbi.1004426)
Supplement: S1 Text — Details of curation of pathways and conversion to logical model. Analysis of model compliance with literature-derived signal transduction data of AGS cells. Cell growth experiments, with dose-response curves for each of the seven inhibitors, and growth curves of 21 combinations of inhibitors. Description of model analysis of FOXO knockouts and its effect on predicted synergies. (PDF) [file pcbi.1004426.s001.pdf]

Supporting information

Table of Contents

Section I – Signaling network building and manual curation 2
1.1 Identification of pathways to include in the manually curated model 2
1.2 Manual curation of the signal transduction model 2
1.2.1 Inclusion of signaling components 2
1.2.2 CellDesigner model 3
1.2.3 Exclusion of signaling components 3
1.2.4 Final SBML signaling model 3
Section II – The Boolean model – definition 4
1.3 Boolean equations for the model of signal transduction in AGS cells 4
1.3.1 Default logical functions 4
1.3.2 Exceptions to the default logical functions 4
Section III - The Boolean model – calibration and validation 5
1.4 Literature review for determination of model component activity state 5
1.5 Literature review of AGS signal transduction perturbation 6
1.6 Model validation 6
Section IV – Model reduction and dual interaction from p38alpha to Antisurvival 8
1.7 Model reduction 8
1.8 Dual interaction from p38alpha to Antisurvival 8
Section V – Simulations of FOXO knockout 8
Section VI – Cell growth experiments 10
1.9 Chemical inhibitors 10
1.10 Synergy assessment 15
1.10.1 Experimental determination of GI50 15
Appendix A 22
Appendix B 22
Appendix C 22
Supplementary references 23

Section I – Signaling network building and manual curation

1.1 Identification of pathways to include in the manually curated model

The AGS gastric adenocarcinoma cell line was chosen because AGS cells harbor mutations in numerous genes encoding proteins that are central in signaling pathways known to be affected in gastric adenocarcinomas: MAPK-, PI3K-, Wnt/β-catenin and NFκB-pathways [55,56,69–71]. These four signaling pathways were chosen as a focus for our signal transduction model. Mutations reported in AGS cells can be found in the databases listed in Table 1.

Table 1: Databases that report mutations in AGS cells.

Database	URL	Reference
Cancer Cell Line Encyclopedia	http://www.broadinstitute.org/ccle/home	[25]
Catalogue of Somatic Mutations in Cancer (COSMIC)	http://cancer.sanger.ac.uk/cancergenome/projects/cosmic/	[24,72]
Genomics of Drug Sensitivity in Cancer	http://www.cancerrxgene.org/	[27]

1.2 Manual curation of the signal transduction model

1.2.1 Inclusion of signaling components

In order to construct a model depicting current knowledge on the MAPK, PI3K, Wnt/β-catenin and NF-κB pathways we used publicly available models in databases as guidance for inclusion of signaling components and interactions. Databases consulted are shown seen in Table 2.

Table 2: Databases consulted for guidance on manual curation.

Database	URL	References
Biocarta	http://www.biocarta.com/	[73]
NetPath	http://www.netpath.org/	[74]
PantherDB	http://www.pantherdb.org/	[75]
Path2Models	https://www.ebi.ac.uk/biomodels-main/path2models	[76]
Pathway Commons	http://www.pathwaycommons.org/	[77]
Pathway Interaction Database	http://pid.nci.nih.gov/	[78]
KEGG	http://www.genome.jp/kegg/	[79,80]
Reactome	http://www.reactome.org/	[81–83]
Science Signaling	http://stke.sciencemag.org/cm/	

All interactions included into the model were substantiated by references to literature documenting experimental evidence for the interaction. In addition, in depth literature review was used to enrich the model with experimentally documented details concerning modulating interactions with the chosen

signaling components and crosstalk between signaling pathways, as well as documented downstream interactions with pro- and anti-survival signaling modules. All literature references are given in Appendix A.

1.2.2 CellDesigner model

The collected pathway information was encoded in a CellDesigner SBML model [84,85] where edges are annotated with literature references from which the interactions are substantiated, see Figure 1. All proteins are annotated with the following information: Uniprot ID, gene name, presence at transcript level in AGS, and presence of mutations in AGS cells as reported in the databases listed in Table 1.

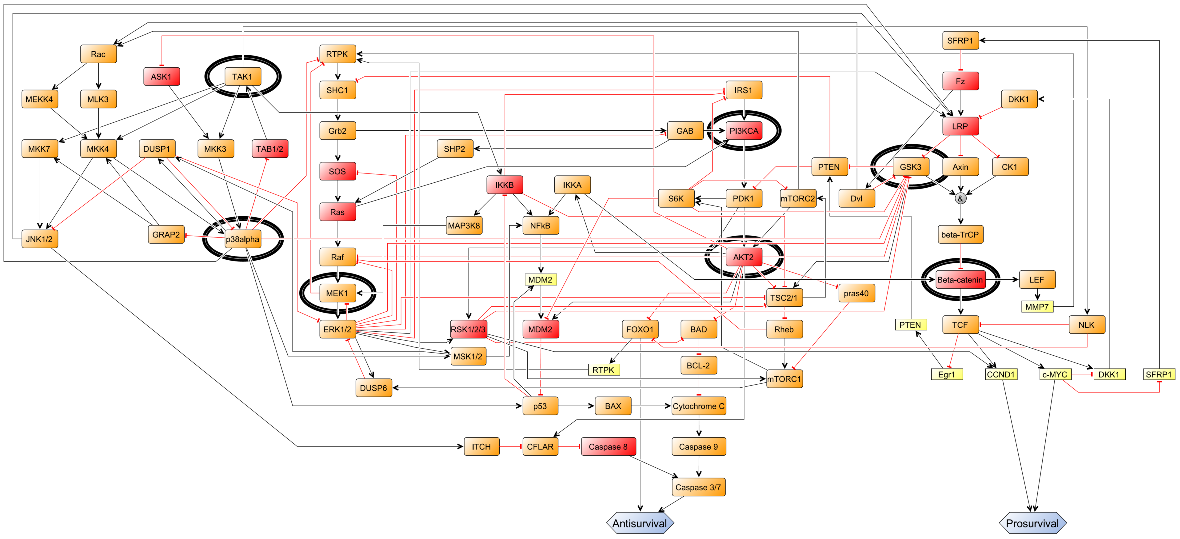

Figure 1: SBML model. Signaling network depicting signal transduction pathways related to growth of AGS gastric adenocarcinoma cells. Black arrows represent activating interactions, while red T arcs represent inhibitory interactions. Yellow boxes denote signal transduction components, and red boxes denote proteins whose gene harbor known mutations in AGS cells. Components identified by black circles mark the components targeted with specific inhibitors. The two diamond-shaped boxes "*Prosurvival*" and "*Antisurvival*" correspond to the two main readouts of the model. The curated model and its annotations are available in SBML format (S1 Dataset).

1.2.3 Exclusion of signaling components

The curated model was trimmed so that only nodes that are regulated by other nodes also present in the model were retained. Thus all model nodes are part of feedback loops, except for the two output nodes and some of their close upstream components (BAD, BAX, BCL2, Caspase3/7, Caspase8, Caspase9, CFLAR, CytochromeC and ITCH).

1.2.4 Final SBML signaling model

The final SBML signaling network consisted of 75 signaling components and two phenotypic output nodes, and 149 interactions, and the full SBML model with annotations can be found in S1 Dataset.

Section II – The Boolean model – definition

1.3 Boolean equations for the model of signal transduction in AGS cells

1.3.1 Default logical functions

We formulated a set of Boolean equations to formalize the evolution rules of each component. Boolean equations were formulated by the following general formula: A signaling component is active if any of its regulatory activators were active, while at the same time none of its regulatory inhibitors were active. For activators *B*, *C* and *D* and inhibitors *E*, *F* and *G* of the node *A*, this translates to:

A = (B or C or D) and not (E or F or G)

Note that several nodes only have activating regulators, and several nodes only have inhibitory regulators.

As described in the main text a few exceptions to this rule were made. The logical formulae of all nodes can be found in S5 Table.

1.3.2 Exceptions to the default logical functions

1.3.2.1 Adjustments to comply with knowledge on protein activity regulation

The general Boolean equation stated above does not always comply with the biological understanding of protein activity regulation. Two Boolean rules were changed because of mechanistic knowledge on the protein activity regulation: 1) The equation describing the activation of the β-catenin destruction complex, which activates β-TrCP, resulting in degradation of β-catenin, and 2) the equation describing β-catenin activity.

For the β-catenin destruction complex to be active all subunits must be available, and so this multi-protein complex was said to depend on all three of Axin, GSK3 and CK1 (note that APC was excluded from the model as it wasn't regulated by any component present in the model, and also note that Dvl was modelled as an intermediary between the Frizzled receptor and GSK3 [86], and not as part of the destruction complex). The logical function is:

betaTrCP = *Axin* and *GSK3* and *CK1*

instead of

betaTrCP = Axin or GSK3 or CK1

The activity of β-catenin should further be governed by β-TrCP in a way so that the absence of β-TrCP causes β-catenin to be active. In the model IKKA is also positively regulating β-catenin activity, and the activity of β-catenin can, if IKKA is active, be sustained even when β-TrCP is active, since IKKA is phosphorylating and protecting β-catenin from destruction [64]. Thus, the equation describing activity of β-catenin was changed from the general formula:

betacatenin = *IKKA* or not *betaTrCP*

instead of

betacatenin = IKKA and not betaTrCP

1.3.2.2 Adjustments to comply with reported observations on protein activity in AGS cells

Seven Boolean rules were adjusted to reflect observations on AGS steady state signaling as reported in publications:

TCF should be active in proliferating AGS cells [55,66], and thus the Boolean formula describing TCF activity was modified:

TCF = betacatenin or not *NLK*

instead of

TCF = betacatenin and not NLK

The logical functions describing the activity of SHC1, SOS, Raf, MEK and ERK were also redefined from the general parameterization by computing inhibitory regulators with OR NOT instead of AND NOT. The impact of negative feedback loops within the SOS/MEK/ERK pathway were thus reduced, replacing the cyclical attractor (in which ERK was cycling) by a single stable state in the model, hence matching published experimental observations for AGS cells, in which ERK is active [65,66]

Table 3: List of Boolean equations that were modified in the ERK pathway.

**General formula**	**Modified**
SHC1 = RTPK and not PTEN	SHC1 = RTPK or not PTEN
SOS = GRB2 and not ERK	SOS = GRB2 or not ERK
MEK = (Raf or MAP3K) and not ERK	MEK = (Raf or MAP3K8) or not ERK
ERK = MEK and not DUSP6	ERK = MEK or not DUSP6
Raf = Ras and not (Rheb or AKT or ERK)	Raf = Ras or not (Rheb or AKT or ERK)
TCF = betacatenin and not NLK	TCF = betacatenin or not NLK

Section III - The Boolean model – calibration and validation

1.4 Literature review for determination of model component activity state

The model protein activities were determined based on a literature review of experimental investigations of proliferating AGS cells. We identified a total of 219 experimental protein state observations relevant for our model reported in 72 unique papers (specified in Appendix B). Most observations were from Western blots, but we also included some reports based on PCR (polymerase chain reaction), EMSA (electrophoretic mobility shift assay), IP (immunoprecipitation), flow cytometry, LC-ES-IT (liquid chromatography electrospray/ionization trap), IHC (immunohistochemistry) or fluorescent reporter proteins. All observations are listed in S2 Table.

Literature reports of protein activity levels were found for 46 of 75 model signal transduction components. We reasoned that whenever the information from several scientific publications consistently implied that a specific protein should be active or inactive in proliferating AGS cells (for example AKT was consistently found to be active), the observed stable state attractor should reflect this (the component AKT value should equal 1). For 21 of the 46 components several, independent and consistent reports were found, and

these activity state values were used for guiding and validating the Boolean equations. Of the 25 component observations that were less well substantiated, ten were found to be in conflict with the predicted state in the attractor. We consider compliance with the 21 well documented components to be of highest importance and therefore concluded that the stable state of the model adequately matches experimental observations of protein activities in actively growing AGS cells.

In searching PubMed for relevant articles, we initially searched for all AGS cell papers reporting Western blots published after the year 2000. This search resulted in 376 papers (the literature survey was performed in June-September 2013). For model proteins with none or only few observations in these initially retrieved papers, a second round of PubMed searches was done retrieving all papers that could be found with the keywords "AGS [missing protein]". This search was not restricted by the time of publication. In addition, Google was used to search for papers with keywords "AGS [missing protein]", identifying a few articles that were not returned by PubMed's internal search engine (even though the paper was indexed by PubMed).

For all observations their suitability was assessed according to the quality of the reported Western blots (subjective perception), specifications of whether cells were in complete medium or starved, whether phosphorylation events or only total protein levels were reported, and if the observation agreed with observations in other papers. An assessment of whether a protein was active or not was then done and activity levels were dichotomized into the two states ON or OFF. See S2 Table.

1.5 Literature review of AGS signal transduction perturbation

As an additional test of the validity of the model, the asymptotic behavior of the *perturbed* model was verified by comparing it with the behavior observed in experimental perturbations. We retrieved information from 56 published experiments where specific signaling proteins had been activated or inhibited (S6 Table), and recorded the reported effects of these perturbations on signaling proteins. The experimentally observed effects were then compared with the effects found for the corresponding nodes in model simulated perturbations. The model accurately reproduced 21 published observations. None of the 56 observations were contradicted, meaning that the model never proposed the result *opposite* to findings in published reports. For 35 observations, the reported change in activity was not captured by the logical model. We consider this to be related to the likelihood that not all activity changes observable on a continuous scale by methods like Western blot will translate into a qualitative signaling change.

In order to capture signal transduction dynamics, the literature was surveyed for reports on AGS cell perturbation experiments where the target was a node in our curated model, and the observed perturbation effect pertained to another node in our model. The papers retrieved from the following PubMed-searches, "AGS siRNA" (163 papers), "AGS [relevant specific inhibitor]", and "AGS inhibitor", were assessed according to the criteria specified above and resulted in 56 observations spanning 20 unique publications that were of relevance to the model (see appendix C).

1.6 Model validation

The predicted stable state of the model fit data from literature, as seen in Table 4. This list of 21 components is a summary of the observations shown in S2 Table, with only the observations of highest confidence included.

Table 4: Model fit with literature data. In the left column the most convincing consensus observations are shown, and contrasted with the model stable state in the right lane.

Protein	Literature	Model
AKT	On	1
Bax	Off	0
BCL2	On	1
β-catenin	On	1
Caspase3/7	Off	0
Caspase8	Off	0
CCND1	On	1
cMYC	On	1
ERK1/2	On	1
GSK3	Off	0
JNK1/2	Off	0
MMP	On	1
NFkB	On	1
p38	Off	0
p53	Off	0
PI3K	On	1
PTEN	Off	0
Rac	On	1
Ras	On	1
S6K	On	1
TCF	On	1

An extended table, where all literature reports are contrasted with model predictions, can be seen in S3 Table. In S3 Table strongly colored components (red/green) are substantiated by several independent and consistent literature observations, and are the same components as listed in Table 4. Less strongly colored components (light red/green) in S3 Table are reported in few papers or with inconsistently reported observations among papers. Yellow colored components have been reported to show both an ON and an OFF state within the same experiment, and for grey colored components there are no published experiments available. Most literature derived observations are coherent with the model, albeit with some exceptions for a few of the less confident literature observations, but as the quality and/or consistency across independent reports for these observations was substantially lower than the observations in Table 4 these discrepancies were not analyzed further.

Section IV – Model reduction and dual interaction from p38alpha to Antisurvival

1.7 Model reduction

Using the built-in model reduction tool of GINsim a logical model can be reduced to a model consisting of fewer nodes, while stable states of the original logical model are preserved. During model reduction, all logical equations are replaced by logical parameters, which amounts to having the logical rules in a lengthy normal disjunctive form. These are provided in S7 Table for the reduced logical model depicted in Figure 3 in the manuscript, along with the reduced model in S3 Dataset. S7 Table was not used for drug synergy analysis, but can provide details to the interactions in model. Compressed expressions of the logical parameters are provided in S8 Table. For each interaction S7 Table specifies the context determining if the interaction is positive, neutral or negative. The context is a discrete vector containing the activity values of all nodes contributing to the nature of the interaction. We use it here to describe the context-dependent nature of the dual interaction from p38alpha to Antisurvival.

1.8 Dual interaction from p38alpha to Antisurvival

After model reduction the interaction from node 'p38alpha' to 'Antisurvival' is shown as 'dual' (Figure 3, Manuscript and S7 Table), where p38alpha can increase the value of 'Antisurvival' in some contexts, and decrease it in other contexts. See S7 Table for a full list of contexts that affects the interaction from p38alpha to Antisurvival. From S7 Table it can be seen that two scenarios can hypothetically happen:

(1) p38alpha inhibition can decrease Antisurvival if AKT is inactive

(2) p38alpha inhibition can increase Antisurvival if AKT is active

Scenario (1) is never observed in our simulations. For (1) to happen one of the drugs in a drug combination would have to cause p38alpha to be activated as p38alpha is inactive in the unperturbed case, and AKT to become inactivated (AKT is active in the unperturbed case). Then if p38alpha inhibition is the second drug in the drug pair we could observe behavior (1). However, among the drug inhibitions we simulated no single drug has the ability to both activate p38alpha and inactivate AKT, and therefore (1) is never observed.

Section V – Simulations of FOXO knockout

From model simulations it was hypothesized that FOXO is important to the predicted and validated synergies. Model simulations where FOXO was forced to be inactive (i.e. knock-out simulations) showed that the synergies of TAK1 and PI3K, and AKT and MEK, and MEK and PI3K were all dependent on FOXO, while the synergy of AKT and MEK was only partially dependent on FOXO (the synergy was weakened but not abolished when FOXO knock-out was simulated). The results from simulations can be seen in Table 5.

Table 5: Simulations of FOXO knock-out reveals that FOXO is necessary to explain the predicted synergies. Node A and B denotes nodes simulated to be inhibited. FOXO indicates whether a FOXO knock-out experiment was simulated (OFF). Prosurvival and Antisurvival show the values of the two phenotypic outputs, which ranges from 0 to 3 for both, and Growth denotes the difference between Prosurvival and Antisurvival, which thus ranges from -3 to 3. In the Synergy column it can be seen that upon FOXO knock-out the synergies of TAK1-PI3K, MEK-PI3K and AKT-TAK1 were abolished, while the synergy of AKT-MEK was still present (albeit weakened). The last column recapitulates the experimental observations of synergies.

Node A	Node B	FOXO	Prosurvival	Antisurvival	Growth	Synergy Model	Synergy Experiment
TAK1	PI3K		2	2	0	1	Yes
TAK1	PI3K	OFF	2	1	1	0	
MEK	PI3K		2	1.5	0.5	0.5	Yes
MEK	PI3K	OFF	2	1	1	0	
AKT	MEK		2.5	2	0.5	1	Yes
AKT	MEK	OFF	2.5	1.5	1	0.5	
AKT	TAK1		3	2	1	1	Yes
AKT	TAK1	OFF	3	1	2	0	
MEK	p38alpha		2	1	1	0.5	No
MEK	p38alpha	OFF	2	1	1	0.5	
PI3K			2	1	1	-	-
PI3K		OFF	2	1	1	-	-
MEK			2	0.5	1.5	-	-
MEK		OFF	2	0.5	1.5	-	
AKT			3	1	2	-	-
AKT		OFF	3	1	2	-	
p38alpha			3	0	3	-	-
p38alpha		OFF	3	0	3	-	
TAK1			3	0	3	-	-
TAK1		OFF	3	0	3	-	
Unperturbed	-		3	0	3	-	-

In addition to the importance of FOXO activation, we found AGS cell growth to be highly dependent on β-catenin, since administration of the β-catenin inhibitor PKF118-310 even at low concentrations profoundly impeded cell growth in comparison with its effect reported for other cell lines [87–89]. Our AGS logical model simulations correlate well with these experimental observations since the growth output exhibited high dependence on the state of the node representing β-catenin, yet model simulations predicted that this trait was not dependent on β-catenin induced activation of FOXO. We thus searched for inhibitor combinations targeting β-catenin and any other node accounted for by the model, to suggest new potent drug combinations. While we found that none of the pairwise inhibitions of components included in the model were able to inactivate β-catenin and simultaneously activate FOXO, model simulations revealed that the strong anti-proliferative state induced by β-catenin inhibition could be further strengthened by simulating activation of FOXO (output values: *Prosurvival* = 0, *Antisurvival* = 2,

contrasted with *Prosurvival* = 0, *Antisurvival* = 1 for β-catenin inhibition alone), emphasizing the central position of FOXO regulation in AGS growth..

Section VI – Cell growth experiments

1.9 Chemical inhibitors

Seven chemical inhibitors were chosen for cell growth experiments. Criteria that guided the selection of inhibitors include:

- Chemical properties similar to clinically approved drugs (Lipinski's rule of five)
- Well characterized in in-vitro kinase screens, in order to know of any off-target effects, and preferentially choose inhibitors with few off-target effects, following the recommendation by the MRC lab whenever possible (http://www.ppu.mrc.ac.uk/).The inhibitors used for perturbation experiments are shown in Table 6.

Table 6: Chemical inhibitors used in perturbation experiments.

**Inhibitor**	**Target**	**Predicted state of target in unperturbed model**	**Dose**
(5Z)-7-oxozeaenol	TAK1	ON	0.5 μM
AKTi-1,2	AKT	ON	10 μM
BIRB0796	p38	OFF	5 μM
CT99021	GSK3	OFF	5 μM
PD0325901	MEK1/2	ON	35 nM
PI103	PI3K	ON	0.7 μM
PKF118-310	β-catenin	ON	150 nM

1.9.1.1 Determination of GI50 concentrations

For each drug a dose-response profile was initially determined, in order to find a dose that inhibited growth by 50%, and which was the dose used in combination experiments. If there was no knowledge on the effective concentration range to be used a 10x dilution series was performed initially, before a 2x dilution series could be done. If the GI50 or a proxy could be found in public databases (Genomics of Drug Sensitivity in Cancer) or deduced from literature (either for AGS cells or other cells) the dilution series would start with a 5x or 2x dilution series.

1.9.1.1.1 Cell growth experiments

All growth measurements were performed in real time, where the xCELLigence RTCA SP/DP (Roche) instrument was used to estimate growth. Real-time measurements of the impedance across the gold arrays at the bottom of wells in a well-plate are reported in the dimensionless unit of cell index which is taken to correspond to cell proliferation.

1.9.1.1.2 Single inhibitors

Figure 2-15 shows the dose response profiles for the seven inhibitors included in the study. In the left lane of figures dose response growth curves versus time for each inhibitor can be seen, and in the right lane of figures the corresponding dose-response profile 48 hours after adding inhibitors is shown. Both the dose-response profile and the dose-response growth curves formed a basis for determining a dose to be used in combination experiments, which should be close to the GI50. DMSO controls were conducted with concentrations matching the highest concentrations used in experiments. In the following paragraphs our results for single inhibitors are compared to literature-derived data.

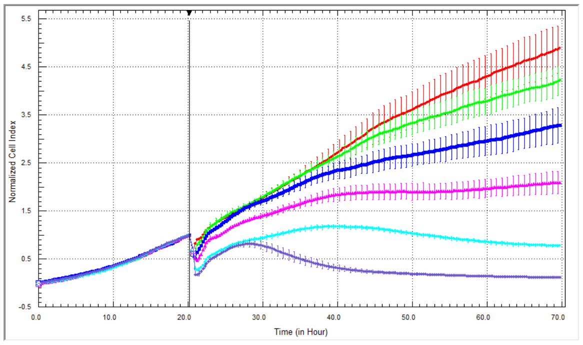

Figure 2: PI103 dose-response. Red: Control. Green: 30 nM. Blue: 150 nM. Pink: 750 nM. Cyan: 3750 nM. Purple: 18.75 µM.

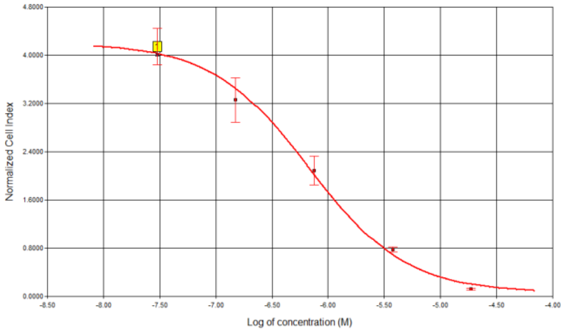

Figure 3: PI103 dose-response. Estimated GI50 is 0.683 µM.

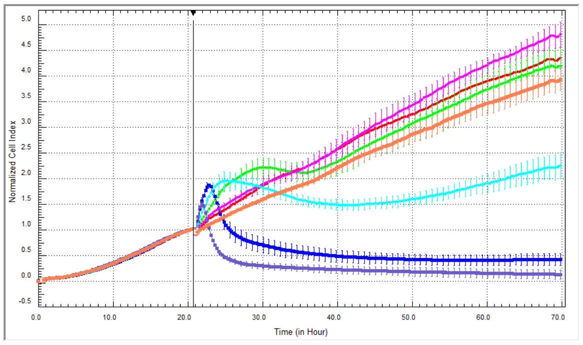

Figure 4: PKF118-310 dose-response. Beige: Control. Red: 44 nM. Pink: 67 nM. Green: 100 nM. Cyan: 150 nM. Blue: 225 nM. Purple: 337.5 nM.

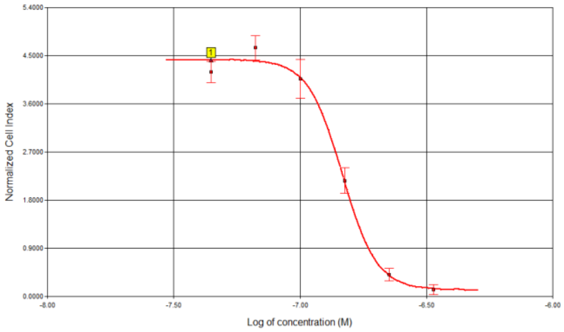

Figure 5: PKF118-310 dose-response. Estimated GI50 is 148 nM.

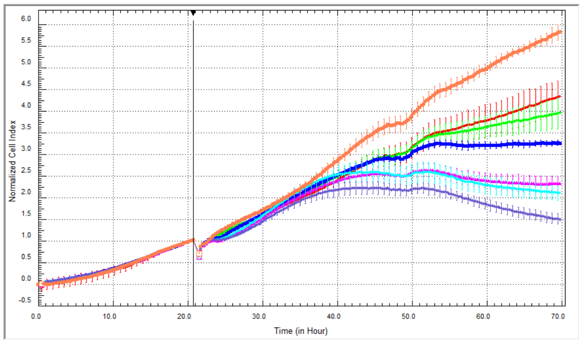

Figure 6: PD0325901 dose-response. Beige: Control. Red: 0.8 nM. Green: 4 nM. Blue: 20 nM. Pink: 100 nM. Cyan: 500 nM. Purple: 2500 nM.

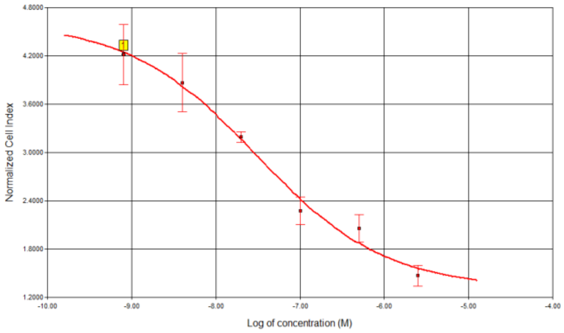

Figure 7: PD0325901 dose-response. Estimated GI50 is 31 nM.

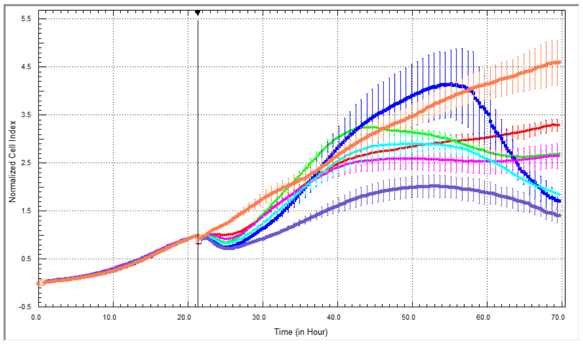

Figure 8: (5Z)-7-oxozeaenol dose response. Beige: Control. Red: 125 nM. Pink: 250 nM. Green: 500 nM. Cyan: 1 µM. Purple: 2 µM.

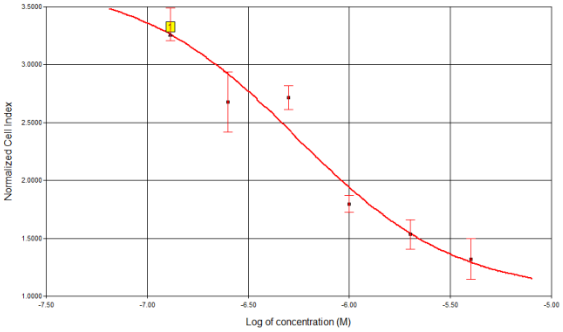

Figure 9: (5Z)-7-oxozeaenol dose response. Estimated GI50 is 0.55 µM.

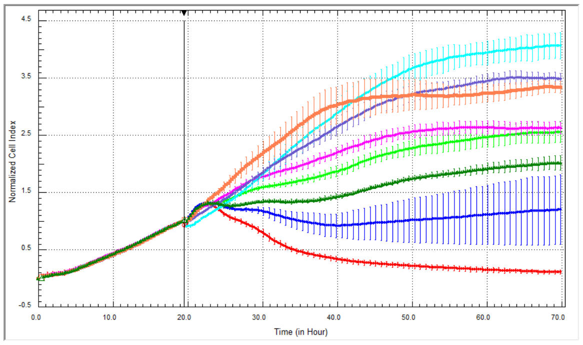

Figure 10: AKTi-1,2 dose-response. Cyan: control. Purple: 2 µM. Beige: 4 µM. Green: 8 µM. Dark green: 12 µM. Blue: 16 µM. Red: 32 µM.

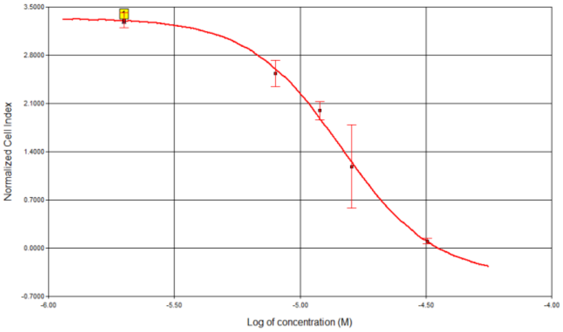

Figure 11: AKTi-1,2 dose response. Estimated GI50 is 10.0 µM. Note that the 8 µM wells in Figure 10 were removed for the estimation on GI50.

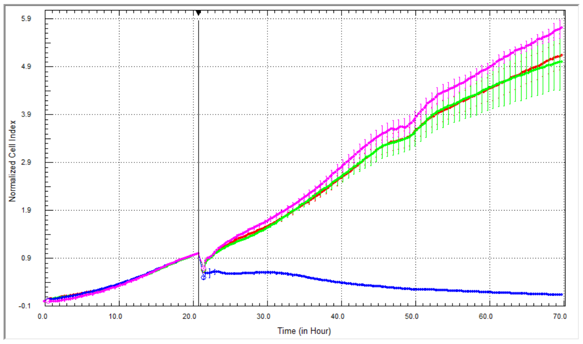

Figure 12: CT99021 dose response. Pink: control. Red: 0.5 µM. Green: 5 µM. Blue: 50 µM.

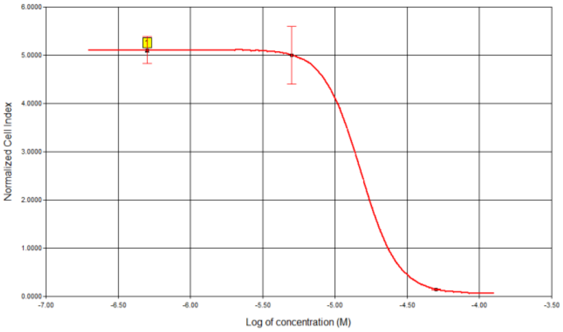

Figure 13: CT99021 dose response. Estimated GI50 is 15.3 µM.

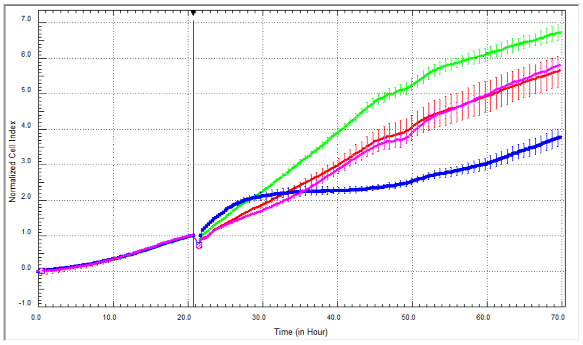

Figure 14: BIRB0796 dose response. Pink: control. Red: 0.5 µM. Green: 5 µM. Blue: 50 µM. As seen in the figure there is an increased growth for cells subjected to BIRB0796 at 5 µM, indicating that AGS cells are insensitive to growth inhibition by BIRB0796.

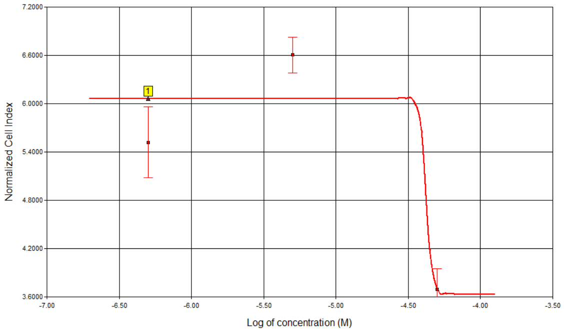

Figure 15: BIRB0796 dose response. Estimated GI50 is 42.3 µM.

1.9.1.2 Observed GI50 values for inhibitors acting on AGS cells

1.9.1.2.1 PI3K inhibitor

The PI3K inhibitor PI103 was found to have a GI50 of 0.7 µM for AGS cells, which fit data from the literature, where the GI50 of PI103 for AGS cells has been reported to be 0.76 µM[90].

1.9.1.2.2 β-catenin inhibitor

The β-catenin inhibitor PKF118-310 was highly potent in AGS cells, and proved to be very difficult to work with in experiments, as there was either an all-or-nothing response for a given concentration. 150 nM was used as an estimate of the GI50, even though this would typically give a full effect. (At 100 nM there would typically be seen no effect). In other cells the GI50 value has been found in the range 0.31-3.48 µM[87–89], indicating that AGS cells are indeed very sensitive to this inhibitor.

1.9.1.2.3 MEK inhibitor

The MEK inhibitor PD0325901 was found to have a GI50 of roughly 35 nM. This fits data from the Genomics of Drug Sensitivity in Cancer database, where the GI50 for AGS cells has been found to be 0.013-0.032 µM.

1.9.1.2.4 TAK1 inhibitor

The TAK1 inhibitor (5Z)-7-oxozeaenol was found to have a GI50 of 0.5 µM in AGS cells. In literature the GI50 for other cell lines has been reported in the range 0.02-60 µM [91,92]. In KRAS-dependent colon cancer cell lines concentrations in the range 0.625-1.25 µM of (5Z)-7-oxozeaenol has been found to promote apoptosis[92], and in another work AGS cells have been described as KRAS dependent[65], and the GI50 found in our experiments are thus in agreement with the few reports of the effects of (5Z)-7-oxozeaenol on proliferation in cell line experiments.

1.9.1.2.5 AKT inhibitor

The AKT inhibitor AKTi-1,2 (AKT inhibitor VIII) was found to have a GI50 in AGS cells of 10 µM. This fits data from the Genomics of Drug Sensitivity in Cancer database, where the GI50 for AGS cells is estimated to be 7.6 µM.

1.9.1.2.6 GSK3 and p38alpha inhibitors

For the two inhibitors CT99021 and BIRB0796 calculated GI50 values are included for reference. This GI50 value is however probably not biologically meaningful as the concentration of each inhibitors is very high compared to concentrations that are typically used to inhibit their intended target. This also coincides with data from the Genomics of Drug Sensitivity in Cancer database, where the GI50 for these inhibitors is estimated by extrapolation to be > 200 µM for AGS cells.

1.9.1.2.7 GI50 vs time

The GI50 varied with time, and so an initial experiment was performed to find a time point at which the GI50 could be estimated. The GI50 stabilized for all inhibitors and combinations of inhibitors within 48 hours after addition of inhibitors, and so this was chosen as the time point for further analysis (both single drug dose response curves, and for drug combination experiments). All growth curves were still recorded in real time, and the 48h time point was used for calculating GI50s and combinatorial indexes (see below). Figure 16 and Figure 17 show two examples of GI50 estimates versus time.

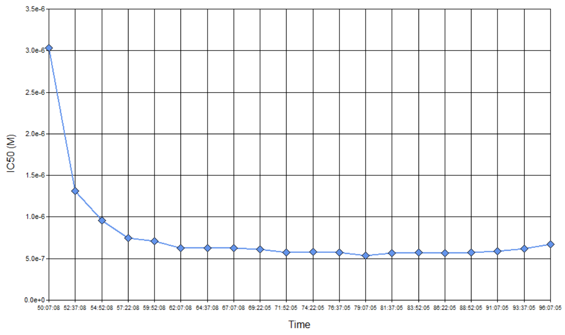

Figure 16: GI50 for PI103 vs time (48h-96h).

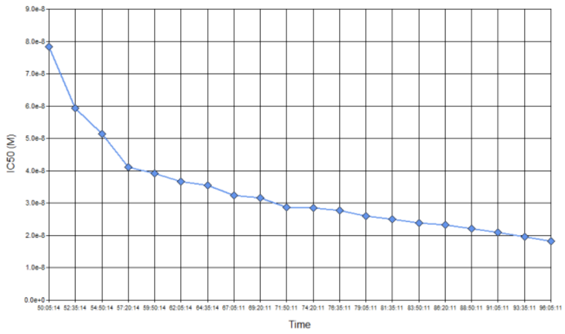

Figure 17: GI50 for PD0325901 vs time (48h-96h)

1.10 Synergy assessment

1.10.1 Experimental determination of GI50

In cell growth experiments the Loewe additivity definition of synergy was used [28]. Initially, a dose-response experiment of each inhibitor was performed to find a concentration of each inhibitor that inhibited growth of cells by half compared to the control (no inhibitor). This dose of the drug is the GI50 (growth inhibition 50%), and for combination experiments a dose close to but less than the GI50 was sought. Since the exact effect of all drugs will vary slightly between biological replicates, experiments

were set up so that both single drugs and their combinations would be tested within one experiment, i.e. within the same biological replicate.

1.10.1.1 Experimental design

All single drugs were tested in three doses within one experiment, in a 2-fold dilution series from the GI50 dose. Similarly the combinations were tested in three doses within one experiment. The highest dose was obtained by mixing the two inhibitors with equal volumes from the GI50 concentration solutions, and thus the concentration of each inhibitor in the combination was half of the GI50. Then a 2-fold dilution series was made so that the doses of each inhibitor were a quarter and an eight of their GI50 doses, which would give three doses where synergy could be assessed according to the Loewe definition. Note that the highest dose of inhibitors in a combination were created from the same aliquots as the single inhibitors, thus ensuring that the concentration in a combination was halved, even if the exact concentration of the single inhibitor deviated slightly from the GI50 concentration.

1.10.1.2 Combination indexes

A combination index (CI) was also calculated for all combinatorial experiments based on the analysis of Chou and Talalay [29], where the CompuSyn software was used for calculating the CI values [68]. The combinatorial index is based on a mechanistic approach to synergy assessment. A median-effect plot is made from specific dose-response data, where the potency and shape of the dose-response is determined, and thus the entire dose-effect curve is predicted. This analysis also takes into account the steepness of the dose-response curves when the effect of combining halves is analyzed (http://www.combosyn.com/) [68,93]. For analyzing the combination index the effect on growth 48 hours after adding inhibitors were used. The GI50 was found to vary with time, but had reached a stable level at this time, as described in the paragraph "GI50 vs time". Note that a CI value can only be reliably computed if a dose-response relationship to an inhibitor can be found, which made CI value calculations impossible for drugs that target proteins that are inactive in proliferating AGS cells (CT99021 targeting GSK3 and BIRB0796 targeting p38).

1.10.1.2.1 Drug combinations

Figures 18-38 shows one representative biological replicate for all 21 drug combinations of the seven inhibitors: (5Z)-7-oxozeaenol; AKTi-1,2; BIRB0796; CT99021; PD0325901; PI103 and PKF118-310. Figure 18 to Figure 21 show the four synergies found. For all figures the combination of two drugs has half the concentration of each single drug in the combination. If the effect of a combination is more profound than the best-performing single inhibitor the effect is synergistic. The plots show the reported Cell Index, which is proportional to the number of cells, on the y-axis, and the time in hours on the x-axis.

The synergy of inhibiting both PI3K and MEK supports previously published observations, including findings in a murine hematopoietic cell line [30], a breast cancer cell line [31], xenograft models of colorectal carcinomas [33], and colon cancer cell lines [6]. Cancer treatment based on combined inhibition of PI3K and MEK is currently being pursued in several phase 1 clinical trials for advanced solid cancer (including pancreatic, breast, non-small cell lung cancer and colorectal cancer; see for instance clinical trials NCT01347866, NCT01363232, NCT01337765 and NCT00996892).

Similarly, the synergistic effect of combining MEK and AKT inhibitors has been previously shown effective against a panel of lung cancer cell lines [32], as well as against thyroid cancer cell lines [34], and is investigated in several clinical trials (including multiple myeloma, breast, endometrial, colorectal, non-

small cell lung cancer, pancreatic cancer, ovarian cancer; see for instance clinical trials NCT01476137, NCT01138085, NCT01907815 and NCT01333475).

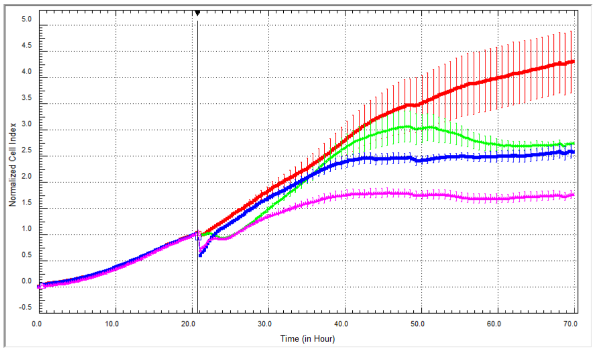

Figure 18: MEK1/2 (PD0325901) and PI3K (PI103) inhibition. PD0325901 35 nM (green) and PI103 0.7 µM (blue) vs their combined effect (pink) and control (red). The combination is synergistic.

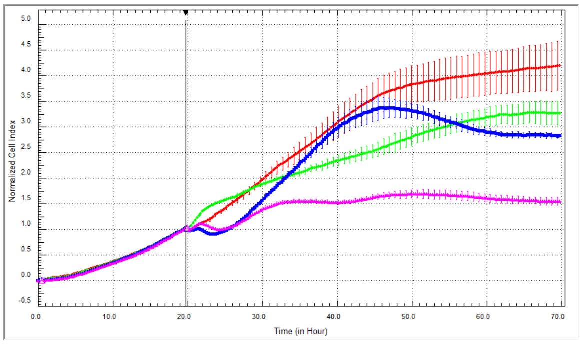

Figure 19: MEK1/2 (PD0325901) and AKT (AKTi-1,2) inhibition. PD0325901 35 nM (blue) and AKTi-1,2 10 µM (green) vs their combined effect (pink) and control (red). The combination is synergistic.

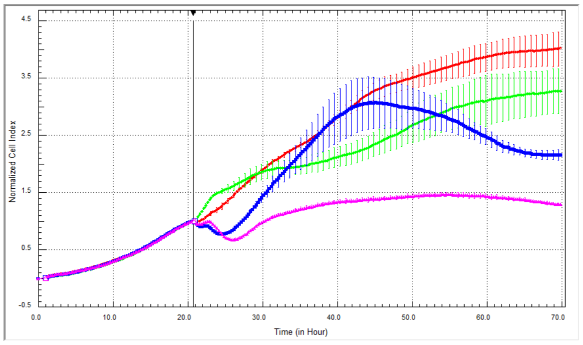

Figure 20: AKT (AKTi-1,2) and TAK1 ((5Z)-7-oxozeaenol) inhibition. AKTi-1,2 10 µM (green) and (5Z)-7-oxozeaenol 0.5 µM (blue) vs their combined effect (pink) and control (red). The combination is synergistic.

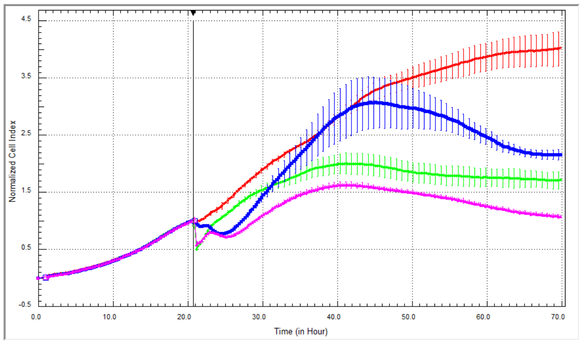

Figure 21: PI3K (PI103) and TAK1 ((5Z)-7-oxozeaenol) inhibition. PI103 0.7 µM (green) and (5Z)-7-oxozeaenol (blue) vs their combined effect (pink) and control (red). The combination is synergistic.

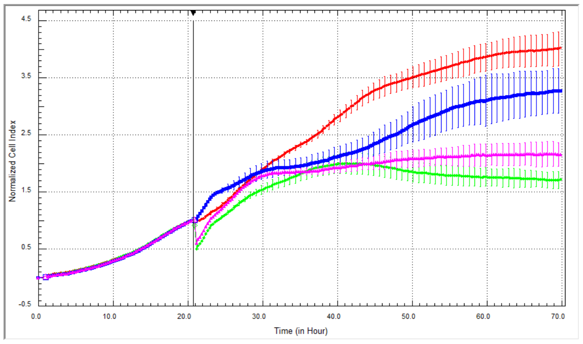

Figure 22: AKT (AKTi-1,2) and PI3K (PI103) inhibition. AKTi-1,2 10 µM (blue) and PI103 0.7 µM (green) vs their combined effect (pink) and control (red). The combination is non-synergistic.

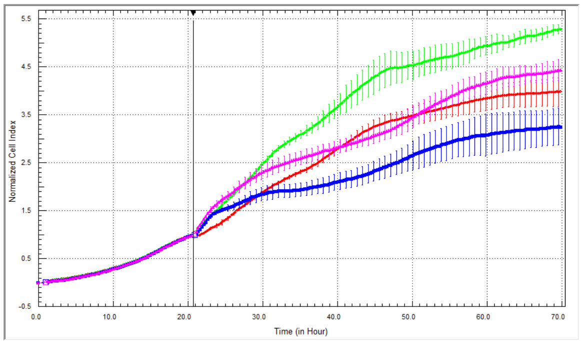

Figure 23: AKT (AKTi-1,2) and p38 alpha (BIRB0796) inhibition. AKTi-1,2 10 µM (blue) and BIRB0796 5 µM (green) vs their combination (pink) and control (red). The combination is non-synergistic.

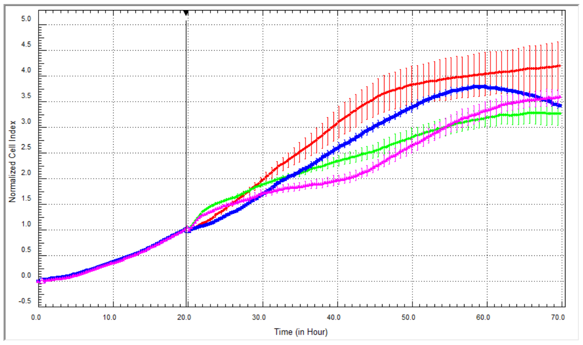

Figure 24: AKT (AKTi-1,2) and GSK3 (CT99021) inhibition. AKTi-1,2 10 µM (green) and CT99021 5 µM (blue) vs their combination (pink) and control (red). The combination is non-synergistic.

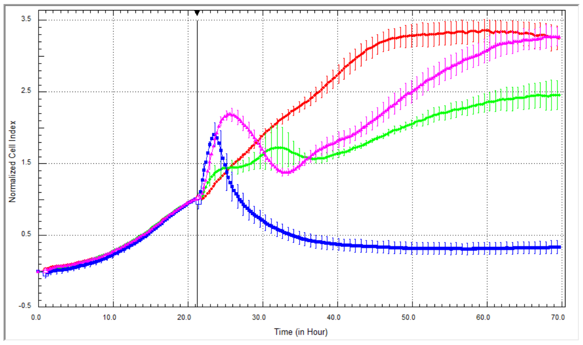

Figure 25: AKT (AKTi-1,2) and β-catenin (PKF118-310) inhibition. AKTi-1,2 10 µM (green) and PKF118-310 150 nM (blue) vs their combination (pink) and control (red). The combination is non-synergistic.

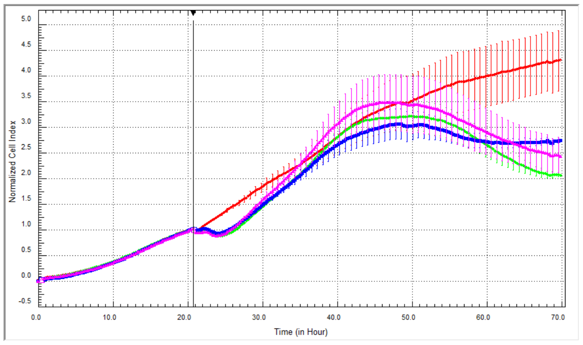

Figure 26: MEK1/2 (PD0325901) and TAK1 ((5Z)-7-oxozeaenol) inhibition. PD0325901 35 nM (blue) and (5Z)-7-oxozeaenol 0.5 µM (green) vs their combination (pink) and control (red). The combination is non-synergistic.

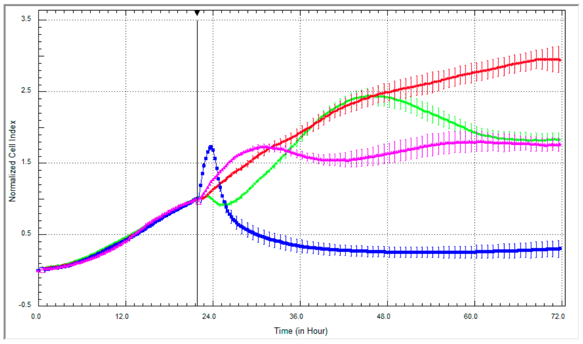

Figure 27: MEK1/2 (PD0325901) and β-catenin (PKF118-310) inhibition. PD0325901 35 nM (green), PKF118-310 150 nM (blue) vs their combination (pink) and control (red). The combination is non-synergistic.

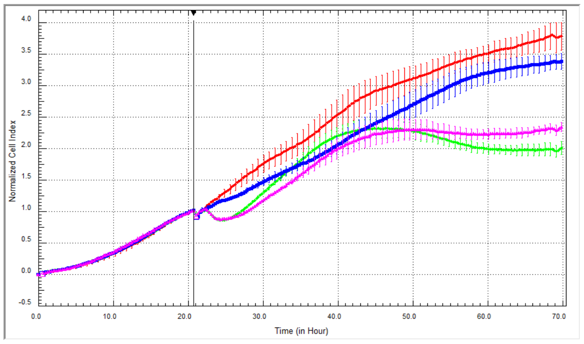

Figure 28: MEK1/2 (PD0325901) and GSK3 (CT99021) inhibition. PD0325901 35 nM (green) and CT99021 5 µM (blue) vs their combination (pink) and control (red). The combination is non-synergistic.

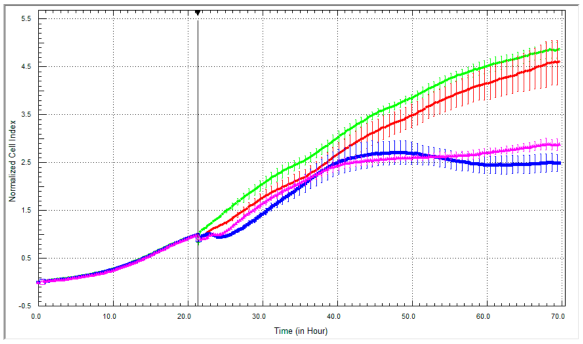

Figure 29: MEK1/2 (PD0325901) and p38 alpha (BIRB0796) inhibition. PD0325901 35 nM (blue) and BIRB0796 5 µM (green) vs their combination (pink) and control (red). The combination is non-synergistic.

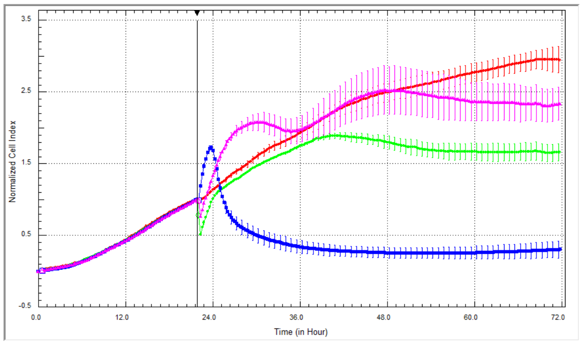

Figure 30: PI3K (PI103) and β-catenin (PKF118-310) inhibition. PI103 0.7 μM (green) and PKF118-310 150 nM (blue) vs their combination (pink) and control (red). The combination is non-synergistic.

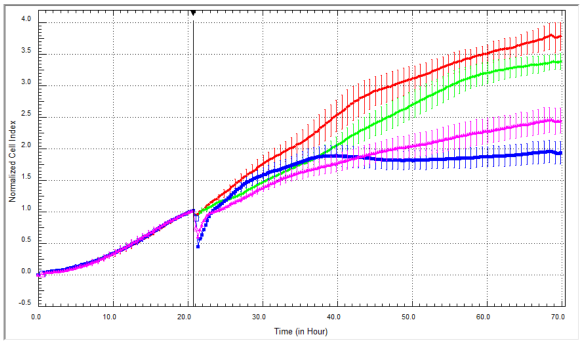

Figure 31: PI3K (PI103) and GSK3 (CT99021) inhibition. PI103 0.7 μM (blue) and CT99021 5 μM (green) vs their combination (pink) and control (red). The combination is non-synergistic.

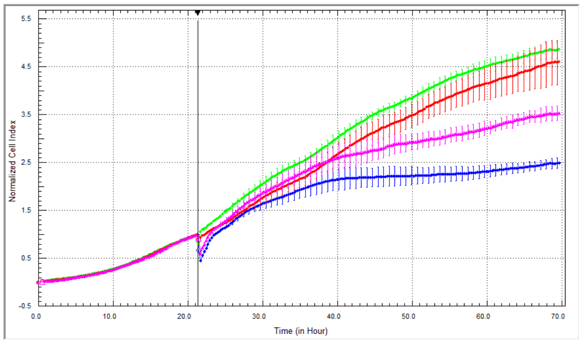

Figure 32: PI3K (PI103) and p38 alpha (BIRB0796) inhibition. PI103 0.7 μM (blue) and BIRB0796 5 μM (green) vs their combination (pink) and control (red). The combination is non-synergistic.

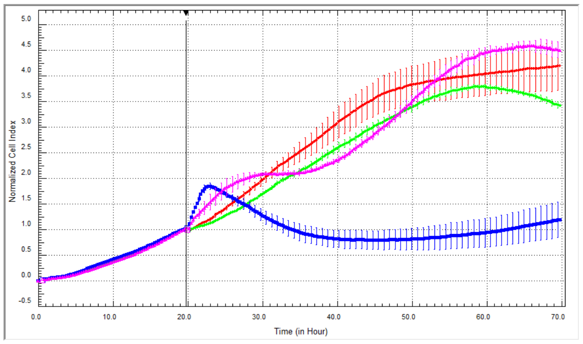

Figure 33: GSK3 (CT999021) and β-catenin (PKF118-310) inhibition. CT99021 5 μM (green) and PKF118-310 150 nM (blue) vs their combination (pink) and control (red).

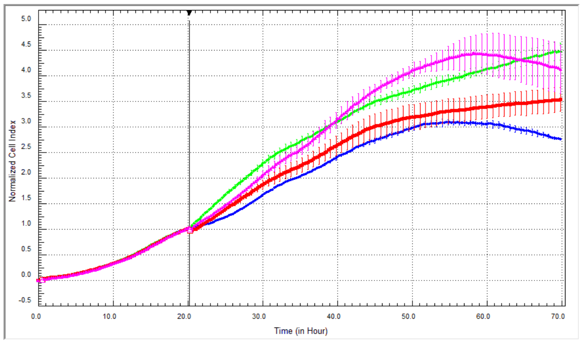

Figure 34: GSK3 (CT99021) and p38 alpha (BIRB0796) inhibition. CT99021 5 μM (blue) and BIRB0796 5 μM (green) vs their combination (pink) and control (red). The combination is non-synergistic.

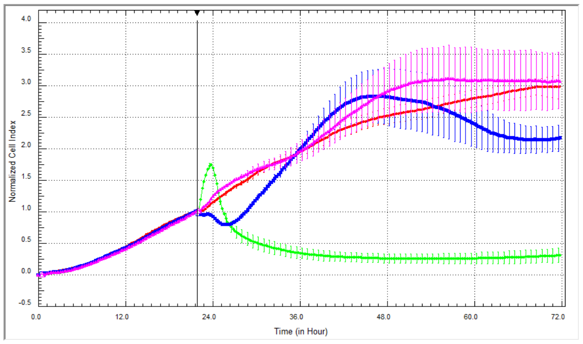

Figure 35: TAK1 ((5Z)-7-oxozeaenol) and β-catenin (PKF118-310) inhibition. (5Z)-7-oxozeaenol 0.5 μM (blue) and PKF118-310 150 nM (green) vs their combination (pink) and control (red). The combination is non-synergistic.

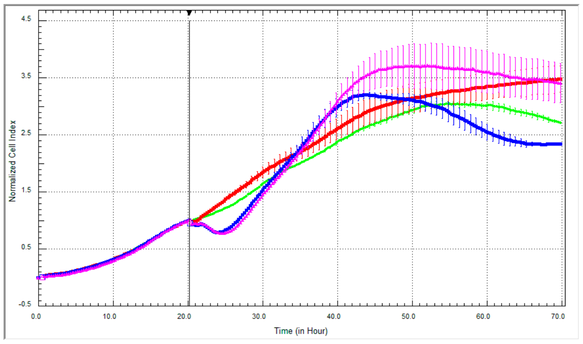

Figure 36: TAK1 ((5Z)-7-oxozeaenol) and GSK3 (CT99021) inhibition. (5Z)-7-oxozeaenol 0.5 µM (blue) and CT99021 5 µM (green) vs their combination (pink) and control (red). The combination is non-synergistic.

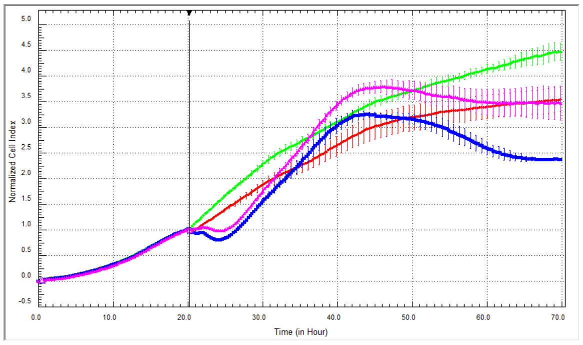

Figure 37: TAK1 ((5Z)-7-oxozeaenol) and p38 alpha (BIRB0796) inhibition. (5Z)-7-oxozeaenol 0.5 µM (blue) and BIRB0796 5 µM (green) vs their combination (pink) and control (red). The combination is non-synergistic.

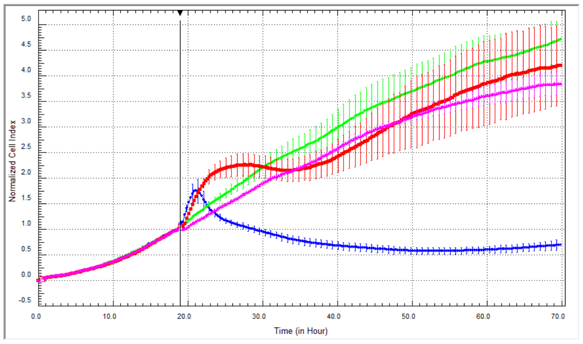

Figure 38: β-catenin (PKF118-310) and p38 alpha (BIRB0796) inhibition. PKF118-310 150 nM (blue) and BIRB0796 5 µM (green) vs their combination (pink) and control (red). The combination is non-synergistic.

Appendix A

Literature references for the curated network model of MAPK-, PI3K, Wnt/β-catenin and NF-κB pathways and their crosstalk (for full reference details, see Supplementary references next page):

[37,38,62,64,86,94–158].

Appendix B

The 218 observations of the state of proteins in AGS cells reported in 71 unique papers (for full reference details, see Supplementary references next page):

[65,66,90,110,146,159–223].

Appendix C

Literature references for the AGS signal transduction perturbation experiments (for full reference details, see Supplementary references next page):

[66,90,159,162,164,166,172,176,178,183,185,189,194,195,197,205,219,224,225].

Supplementary references

69. Kasper S, Schuler M. Targeted therapies in gastroesophageal cancer. European Journal of Cancer. Elsevier Ltd; 2014. pp. 1247–1258. doi:10.1016/j.ejca.2014.01.009

70. De Luca A, Maiello MR, D'Alessio A, Pergameno M, Normanno N. The RAS/RAF/MEK/ERK and the PI3K/AKT signalling pathways: role in cancer pathogenesis and implications for therapeutic approaches. Expert Opin Ther Targets. 2012;16 Suppl 2: S17–27. doi:10.1517/14728222.2011.639361

71. Osaki LH, Gama P. MAPKs and Signal Transduction in the Control of Gastrointestinal Epithelial Cell Proliferation and Differentiation. Int J Mol Sci. 2013;14: 10143–61. doi:10.3390/ijms140510143

72. Forbes SA, Bhamra G, Bamford S, Dawson E, Kok C, Clements J, et al. The Catalogue of Somatic Mutations in Cancer (COSMIC). Curr Protoc Hum Genet. 2008;Chapter 10: Unit 10.11. doi:10.1002/0471142905.hg1011s57

73. Nishimura D. BioCarta. Biotech Softw Internet Rep. 2001;2: 117–120. doi:10.1089/152791601750294344

74. Kandasamy K, Mohan SS, Raju R, Keerthikumar S, Kumar GSS, Venugopal AK, et al. NetPath: a public resource of curated signal transduction pathways. Genome Biol. 2010;11: R3. doi:10.1186/gb-2010-11-1-r3

75. Mi H, Muruganujan A, Thomas PD. PANTHER in 2013: modeling the evolution of gene function, and other gene attributes, in the context of phylogenetic trees. Nucleic Acids Res. 2013;41: D377–86. doi:10.1093/nar/gks1118

76. Büchel F, Rodriguez N, Swainston N, Wrzodek C, Czauderna T, Keller R, et al. Path2Models: large-scale generation of computational models from biochemical pathway maps. BMC Syst Biol. 2013;7: 116. doi:10.1186/1752-0509-7-116

77. Cerami EG, Gross BE, Demir E, Rodchenkov I, Babur O, Anwar N, et al. Pathway Commons, a web resource for biological pathway data. Nucleic Acids Res. 2011;39: D685–90. doi:10.1093/nar/gkq1039

78. Schaefer CF, Anthony K, Krupa S, Buchoff J, Day M, Hannay T, et al. PID: the Pathway Interaction Database. Nucleic Acids Res. 2009;37: D674–9. doi:10.1093/nar/gkn653

79. Kanehisa M, Goto S. KEGG: kyoto encyclopedia of genes and genomes. Nucleic Acids Res. 2000;28: 27–30.

80. Kanehisa M, Goto S, Sato Y, Kawashima M, Furumichi M, Tanabe M. Data, information, knowledge and principle: back to metabolism in KEGG. Nucleic Acids Res. 2014;42: D199–205. doi:10.1093/nar/gkt1076

81. Matthews L, Gopinath G, Gillespie M, Caudy M, Croft D, de Bono B, et al. Reactome knowledgebase of human biological pathways and processes. Nucleic Acids Res. 2009;37: D619–22. doi:10.1093/nar/gkn863

82. Vastrik I, D'Eustachio P, Schmidt E, Joshi-Tope G, Gopinath G, Croft D, et al. Reactome: a knowledge base of biologic pathways and processes. Genome Biol. 2007;8: R39. doi:10.1186/gb-2007-8-3-r39

83. Joshi-Tope G, Vastrik I, Gopinath GR, Matthews L, Schmidt E, Gillespie M, et al. The Genome Knowledgebase: a resource for biologists and bioinformaticists. Cold Spring Harb Symp Quant Biol. 2003;68: 237–43.

84. Funahashi A, Morohashi M, Kitano H, Tanimura N. CellDesigner : a process diagram editor for gene-regulatory and. Biosilico. 2003;1: 159–162.

85. Funahashi A, Matsuoka Y, Jouraku A, Morohashi M, Kikuchi N, Kitano H. CellDesigner 3.5: A Versatile Modeling Tool for Biochemical Networks. Proc IEEE. 2008;96: 1254–1265. doi:10.1109/JPROC.2008.925458

86. Gao C, Chen Y-G. Dishevelled: The hub of Wnt signaling. Cell Signal. Elsevier Inc.; 2010;22: 717–27. doi:10.1016/j.cellsig.2009.11.021

87. Lu W, Tinsley HN, Keeton A, Qu Z, Piazza G a, Li Y. Suppression of Wnt/beta-catenin signaling inhibits prostate cancer cell proliferation. Eur J Pharmacol. Elsevier B.V.; 2009;602: 8–14. doi:10.1016/j.ejphar.2008.10.053

88. Wei W, Chua M-S, Grepper S, So S. Small molecule antagonists of Tcf4/beta-catenin complex inhibit the growth of HCC cells in vitro and in vivo. Int J Cancer. 2010;126: 2426–36. doi:10.1002/ijc.24810

89. Hallett RM, Kondratyev MK, Giacomelli AO, Nixon AML, Girgis-Gabardo A, Ilieva D, et al. Small molecule antagonists of the Wnt/β-catenin signaling pathway target breast tumor-initiating cells in a Her2/Neu mouse model of breast cancer. PLoS One. 2012;7: e33976. doi:10.1371/journal.pone.0033976

90. Bhattacharya B, Akram M, Balasubramanian I, Tam KKY, Koh KX, Yee MQ, et al. Pharmacologic synergy between dual phosphoinositide-3-kinase and mammalian target of rapamycin inhibition and 5-fluorouracil in PIK3CA mutant gastric cancer cells. Cancer Biol Ther. 2012;13: 34–42. doi:10.4161/cbt.13.1.18437

91. Wu J, Powell F, Larsen N a, Lai Z, Byth KF, Read J, et al. Mechanism and in vitro pharmacology of TAK1 inhibition by (5Z)-7-Oxozeaenol. ACS Chem Biol. 2013;8: 643–50. doi:10.1021/cb3005897

92. Singh A, Sweeney MF, Yu M, Burger A, Greninger P, Benes C, et al. TAK1 inhibition promotes apoptosis in KRAS-dependent colon cancers. Cell. Elsevier Inc.; 2012;148: 639–50. doi:10.1016/j.cell.2011.12.033

93. Chou T-C. Drug combination studies and their synergy quantification using the Chou-Talalay method. Cancer Res. 2010;70: 440–6. doi:10.1158/0008-5472.CAN-09-1947

94. Yanai K, Nakamura M, Akiyoshi T, Nagai S, Wada J, Koga K, et al. Crosstalk of hedgehog and Wnt pathways in gastric cancer. Cancer Lett. 2008;263: 145–56. doi:10.1016/j.canlet.2007.12.030

95. Lau M-T, Klausen C, Leung PCK. E-cadherin inhibits tumor cell growth by suppressing PI3K/Akt signaling via β-catenin-Egr1-mediated PTEN expression. Oncogene. Nature Publishing Group; 2011;30: 2753–66. doi:10.1038/onc.2011.6

96. Saegusa M, Hashimura M, Kuwata T, Hamano M, Watanabe J, Kawaguchi M, et al. Transcription factor Egr1 acts as an upstream regulator of beta-catenin signalling through up-regulation of TCF4 and p300 expression during trans-differentiation of endometrial carcinoma cells. J Pathol. 2008;216: 521–32. doi:10.1002/path.2404

97. Zeller E, Hammer K, Kirschnick M, Braeuning A. Mechanisms of RAS/β-catenin interactions. Arch Toxicol. 2013; 611–632. doi:10.1007/s00204-013-1035-3

98. Gustavson MD, Crawford HC, Fingleton B, Matrisian LM. Tcf binding sequence and position determines beta-catenin and Lef-1 responsiveness of MMP-7 promoters. Mol Carcinog. 2004;41: 125–39. doi:10.1002/mc.20049

99. Bryja V, Gradl D, Schambony A, Arenas E, Schulte G. Beta-arrestin is a necessary component of Wnt/beta-catenin signaling in vitro and in vivo. Proc Natl Acad Sci U S A. 2007;104: 6690–5. doi:10.1073/pnas.0611356104

100. Engelman J a. Targeting PI3K signalling in cancer: opportunities, challenges and limitations. Nat Rev Cancer. Nature Publishing Group; 2009;9: 550–62. doi:10.1038/nrc2664

101. Ma XM, Blenis J. Molecular mechanisms of mTOR-mediated translational control. Nat Rev Mol Cell Biol. 2009;10: 307–18. doi:10.1038/nrm2672

102. Mendoza MC, Er EE, Blenis J. The Ras-ERK and PI3K-mTOR pathways: cross-talk and compensation. Trends Biochem Sci. Elsevier Ltd; 2011;36: 320–8. doi:10.1016/j.tibs.2011.03.006

103. Sabatini DM. mTOR and cancer: insights into a complex relationship. Nat Rev Cancer. 2006;6: 729–34. doi:10.1038/nrc1974

104. Laplante M, Sabatini DM. mTOR signaling in growth control and disease. Cell. Elsevier Inc.; 2012;149: 274–93. doi:10.1016/j.cell.2012.03.017

105. Zhang Y, Gan B, Liu D, Paik J. FoxO family members in cancer. Cancer Biol Ther. 2011;12: 253–9. doi:10.4161/cbt.12.4.15954

106. Fu Z, Tindall DJ. FOXOs, cancer and regulation of apoptosis. Oncogene. 2008;27: 2312–9. doi:10.1038/onc.2008.24

107. Brabletz S, Schmalhofer O, Brabletz T. Gastrointestinal stem cells in development and cancer. J Pathol. 2009;217: 307–17. doi:10.1002/path.2475

108. Valenta T, Hausmann G, Basler K. The many faces and functions of β-catenin. EMBO J. 2012;31: 2714–36. doi:10.1038/emboj.2012.150

109. Wu D, Pan W. GSK3: a multifaceted kinase in Wnt signaling. Trends Biochem Sci. Elsevier Ltd; 2010;35: 161–8. doi:10.1016/j.tibs.2009.10.002

110. Choi HS, Seo H-S, Kim JH, Um J-Y, Shin YC, Ko S-G. Ethanol extract of paeonia suffruticosa Andrews (PSE) induced AGS human gastric cancer cell apoptosis via fas-dependent apoptosis and MDM2-p53 pathways. J Biomed Sci. Journal of Biomedical Science; 2012;19: 82. doi:10.1186/1423-0127-19-82

111. Ji H, Ding Z, Hawke D, Xing D, Jiang B-H, Mills GB, et al. AKT-dependent phosphorylation of Niban regulates nucleophosmin- and MDM2-mediated p53 stability and cell apoptosis. EMBO Rep. Nature Publishing Group; 2012;13: 554–60. doi:10.1038/embor.2012.53

112. Wade M, Li Y-C, Wahl GM. MDM2, MDMX and p53 in oncogenesis and cancer therapy. Nat Rev Cancer. 2013;13: 83–96. doi:10.1038/nrc3430

113. Chetram M a, Hinton C V. PTEN regulation of ERK1/2 signaling in cancer. J Recept Signal Transduct Res. 2012;32: 190–5. doi:10.3109/10799893.2012.695798

114. Cotter TG. Apoptosis and cancer: the genesis of a research field. Nat Rev Cancer. Nature Publishing Group; 2009;9: 501–7. doi:10.1038/nrc2663

115. Bratton SB, Salvesen GS. Regulation of the Apaf-1-caspase-9 apoptosome. J Cell Sci. 2010;123: 3209–14. doi:10.1242/jcs.073643

116. Pullen N, Dennis PB, Andjelkovic M, Dufner A, Kozma SC, Hemmings BA, et al. Phosphorylation and activation of p70s6k by PDK1. Science. 1998;279: 707–10. doi:10.1126/science.279.5351.707

117. Bayascas JR. PDK1: the major transducer of PI 3-kinase actions. Curr Top Microbiol Immunol. 2010;346: 9–29. doi:10.1007/82_2010_43

118. Cully M, You H, Levine AJ, Mak TW. Beyond PTEN mutations: the PI3K pathway as an integrator of multiple inputs during tumorigenesis. Nat Rev Cancer. 2006;6: 184–92. doi:10.1038/nrc1819

119. Anjum R, Blenis J. The RSK family of kinases: emerging roles in cellular signalling. Nat Rev Mol Cell Biol. 2008;9: 747–58. doi:10.1038/nrm2509

120. Belov A a, Mohammadi M. Grb2, a double-edged sword of receptor tyrosine kinase signaling. Sci Signal. 2012;5: pe49. doi:10.1126/scisignal.2003576

121. Wills MKB, Jones N. Teaching an old dogma new tricks: twenty years of Shc adaptor signalling. Biochem J. 2012;447: 1–16. doi:10.1042/BJ20120769

122. Červenka I, Wolf J, Mašek J, Krejci P, Wilcox WR, Kozubík A, et al. Mitogen-activated protein kinases promote WNT/beta-catenin signaling via phosphorylation of LRP6. Mol Cell Biol. 2011;31: 179–89. doi:10.1128/MCB.00550-10

123. Beauchamp EM, Platanias LC. The evolution of the TOR pathway and its role in cancer. Oncogene. Nature Publishing Group; 2013;32: 3923–32. doi:10.1038/onc.2012.567

124. Magnuson B, Ekim B, Fingar DC. Regulation and function of ribosomal protein S6 kinase (S6K) within mTOR signalling networks. Biochem J. 2012;441: 1–21. doi:10.1042/BJ20110892

125. Haeusgen W, Herdegen T, Waetzig V. The bottleneck of JNK signaling: molecular and functional characteristics of MKK4 and MKK7. Eur J Cell Biol. Elsevier GmbH.; 2011;90: 536–44. doi:10.1016/j.ejcb.2010.11.008

126. Seki E, Brenner D a, Karin M. A liver full of JNK: signaling in regulation of cell function and disease pathogenesis, and clinical approaches. Gastroenterology. Elsevier Inc.; 2012;143: 307–20. doi:10.1053/j.gastro.2012.06.004

127. Sugimura R, Li L. Noncanonical Wnt signaling in vertebrate development, stem cells, and diseases. Birth Defects Res C Embryo Today. 2010;90: 243–56. doi:10.1002/bdrc.20195

128. Taylor JL, Szmulewitz RZ, Lotan T, Hickson J, Griend D Vander, Yamada SD, et al. New paradigms for the function of JNKK1/MKK4 in controlling growth of disseminated cancer cells. Cancer Lett. Elsevier Ireland Ltd; 2008;272: 12–22. doi:10.1016/j.canlet.2008.05.012

129. Bogoyevitch M a, Kobe B. Uses for JNK: the many and varied substrates of the c-Jun N-terminal kinases. Microbiol Mol Biol Rev. 2006;70: 1061–95. doi:10.1128/MMBR.00025-06

130. Weston CR, Davis RJ. The JNK signal transduction pathway. Curr Opin Cell Biol. 2007;19: 142–9. doi:10.1016/j.ceb.2007.02.001

131. Li M, Wang H, Huang T, Wang J, Ding Y, Li Z, et al. TAB2 scaffolds TAK1 and NLK in repressing canonical Wnt signaling. J Biol Chem. 2010;285: 13397–404. doi:10.1074/jbc.M109.083246

132. Ishitani T, Kishida S, Hyodo-Miura J, Ueno N, Yasuda J, Waterman M, et al. The TAK1-NLK mitogen-activated protein kinase cascade functions in the Wnt-5a/Ca(2+) pathway to antagonize Wnt/beta-catenin signaling. Mol Cell Biol. 2003;23: 131–9. doi:10.1128/MCB.23.1.131

133. Bermudez O, Pagès G, Gimond C. The dual-specificity MAP kinase phosphatases: critical roles in development and cancer. Am J Physiol Cell Physiol. 2010;299: C189–202. doi:10.1152/ajpcell.00347.2009

134. Boutros T, Chevet E, Metrakos P. Mitogen-activated protein (MAP) kinase/MAP kinase phosphatase regulation: roles in cell growth, death, and cancer. Pharmacol Rev. 2008;60: 261–310. doi:10.1124/pr.107.00106

135. Sun S-C. Non-canonical NF-κB signaling pathway. Cell Res. Nature Publishing Group; 2011;21: 71–85. doi:10.1038/cr.2010.177

136. Chen ZJ. Ubiquitination in signaling to and activation of IKK. Immunol Rev. 2012;246: 95–106. doi:10.1111/j.1600-065X.2012.01108.x

137. Harris SL, Levine AJ. The p53 pathway: positive and negative feedback loops. Oncogene. 2005;24: 2899–908. doi:10.1038/sj.onc.1208615

138. Chandarlapaty S, Sawai A, Scaltriti M, Rodrik-Outmezguine V, Grbovic-Huezo O, Serra V, et al. AKT inhibition relieves feedback suppression of receptor tyrosine kinase expression and activity. Cancer Cell. Elsevier Inc.; 2011;19: 58–71. doi:10.1016/j.ccr.2010.10.031

139. Kim AH, Khursigara G, Sun X, Franke TF, Chao M V. Akt phosphorylates and negatively regulates apoptosis signal-regulating kinase 1. Mol Cell Biol. 2001;21: 893–901. doi:10.1128/MCB.21.3.893-901.2001

140. Chang L, Kamata H, Solinas G, Luo J-L, Maeda S, Venuprasad K, et al. The E3 ubiquitin ligase itch couples JNK activation to TNFalpha-induced cell death by inducing c-FLIP(L) turnover. Cell. 2006;124: 601–13. doi:10.1016/j.cell.2006.01.021

141. Slocombe W. Turning the tables. Ment Health Today. 2007;6: 28–9.

142. Cowling VH, D'Cruz CM, Chodosh L a, Cole MD. c-Myc transforms human mammary epithelial cells through repression of the Wnt inhibitors DKK1 and SFRP1. Mol Cell Biol. 2007;27: 5135–46. doi:10.1128/MCB.02282-06

143. Panner A, Crane C a, Weng C, Feletti A, Fang S, Parsa AT, et al. Ubiquitin-specific protease 8 links the PTEN-Akt-AIP4 pathway to the control of FLIPS stability and TRAIL sensitivity in glioblastoma multiforme. Cancer Res. 2010;70: 5046–53. doi:10.1158/0008-5472.CAN-09-3979

144. Safa a R. c-FLIP, a master anti-apoptotic regulator. Exp Oncol. 2012;34: 176–84.

145. Uriarte SM, Joshi-Barve S, Song Z, Sahoo R, Gobejishvili L, Jala VR, et al. Akt inhibition upregulates FasL, downregulates c-FLIPs and induces caspase-8-dependent cell death in Jurkat T lymphocytes. Cell Death Differ. 2005;12: 233–42. doi:10.1038/sj.cdd.4401549

146. Zheng H, Xu X, Xia P, Yu M, Takahashi H, Takano Y. Involvement of inactive GSK3beta overexpression in tumorigenesis and progression of gastric carcinomas. Hum Pathol. Elsevier B.V.; 2010;41: 1255–64. doi:10.1016/j.humpath.2010.02.003

147. Maccario H, Perera NM, Davidson L, Downes CP, Leslie NR. PTEN is destabilized by phosphorylation on Thr366. Biochem J. 2007;405: 439–44. doi:10.1042/BJ20061837

148. Lai KP, Leong WF, Chau JFL, Jia D, Zeng L, Liu H, et al. S6K1 is a multifaceted regulator of Mdm2 that connects nutrient status and DNA damage response. EMBO J. Nature Publishing Group; 2010;29: 2994–3006. doi:10.1038/emboj.2010.166

149. Sparks C a, Guertin D a. Targeting mTOR: prospects for mTOR complex 2 inhibitors in cancer therapy. Oncogene. Nature Publishing Group; 2010;29: 3733–44. doi:10.1038/onc.2010.139

150. Vougioukalaki M, Kanellis DC, Gkouskou K, Eliopoulos AG. Tpl2 kinase signal transduction in inflammation and cancer. Cancer Lett. Elsevier Ireland Ltd; 2011;304: 80–9. doi:10.1016/j.canlet.2011.02.004

151. Gilmore TD. Introduction to NF-kappaB: players, pathways, perspectives. Oncogene. 2006;25: 6680–4. doi:10.1038/sj.onc.1209954

152. Kyriakis JM, Avruch J. Mammalian MAPK signal transduction pathways activated by stress and inflammation: a 10-year update. Physiol Rev. 2012;92: 689–737. doi:10.1152/physrev.00028.2011

153. Lara R, Seckl MJ, Pardo OE. The p90 RSK family members: common functions and isoform specificity. Cancer Res. 2013;73: 5301–8. doi:10.1158/0008-5472.CAN-12-4448

154. Astrinidis A, Henske EP. Tuberous sclerosis complex: linking growth and energy signaling pathways with human disease. Oncogene. 2005;24: 7475–81. doi:10.1038/sj.onc.1209090

155. Karbowniczek M, Robertson GP, Henske EP. Rheb inhibits C-raf activity and B-raf/C-raf heterodimerization. J Biol Chem. 2006;281: 25447–56. doi:10.1074/jbc.M605273200

156. Pierre S, Bats A-S, Coumoul X. Understanding SOS (Son of Sevenless). Biochem Pharmacol. 2011;82: 1049–56. doi:10.1016/j.bcp.2011.07.072

157. Castellano E, Downward J. RAS Interaction with PI3K: More Than Just Another Effector Pathway. Genes Cancer. 2011;2: 261–74. doi:10.1177/1947601911408079

158. Lambert JM, Lambert QT, Reuther GW, Malliri A, Siderovski DP, Sondek J, et al. Tiam1 mediates Ras activation of Rac by a PI(3)K-independent mechanism. Nat Cell Biol. 2002;4: 621–5. doi:10.1038/ncb833

159. Aquilano K, Baldelli S, Rotilio G, Ciriolo MR. trans-Resveratrol inhibits H2O2-induced adenocarcinoma gastric cells proliferation via inactivation of MEK1/2-ERK1/2-c-Jun signalling axis. Biochem Pharmacol. 2009;77: 337–47. doi:10.1016/j.bcp.2008.10.034

160. Redlak MJ, Miller T a. Targeting PI3K/Akt/HSP90 signaling sensitizes gastric cancer cells to deoxycholate-induced apoptosis. Dig Dis Sci. 2011;56: 323–9. doi:10.1007/s10620-010-1294-2

161. Patel O, Marshall KM, Bramante G, Baldwin GS, Shulkes A. The C-terminal flanking peptide (CTFP) of progastrin inhibits apoptosis via a PI3-kinase-dependent pathway. Regul Pept. Elsevier B.V.; 2010;165: 224–31. doi:10.1016/j.regpep.2010.08.005

162. Liu J, Fu X-Q, Zhou W, Yu H-G, Yu J-P, Luo H-S. LY294002 potentiates the anti-cancer effect of oxaliplatin for gastric cancer via death receptor pathway. World J Gastroenterol. 2011;17: 181–90. doi:10.3748/wjg.v17.i2.181

163. Vangamudi B, Zhu S, Soutto M, Belkhiri A, El-Rifai W. Regulation of β-catenin by t-DARPP in upper gastrointestinal cancer cells. Mol Cancer. BioMed Central Ltd; 2011;10: 32. doi:10.1186/1476-4598-10-32

164. Tseng P-C, Huang W-C, Chen C-L, Sheu B-S, Shan Y-S, Tsai C-C, et al. Regulation of SHP2 by PTEN/AKT/GSK-3β signaling facilitates IFN-γ resistance in hyperproliferating gastric cancer. Immunobiology. Elsevier GmbH.; 2012;217: 926–34. doi:10.1016/j.imbio.2012.01.001

165. Xu X, Xia P, Yu M, Nie X, Yang X, Xing Y, et al. The roles of REIC gene and its encoding product in gastric carcinoma. Cell Cycle. 2012;11: 1414–31. doi:10.4161/cc.19823

166. Mueller A, Bachmann E, Linnig M, Khillimberger K, Schimanski CC, Galle PR, et al. Selective PI3K inhibition by BKM120 and BEZ235 alone or in combination with chemotherapy in wild-type and mutated human gastrointestinal cancer cell lines. Cancer Chemother Pharmacol. 2012;69: 1601–15. doi:10.1007/s00280-012-1869-z

167. Kundu J, Wahab SMR, Kundu JK, Choi Y-L, Erkin OC, Lee HS, et al. Tob1 induces apoptosis and inhibits proliferation, migration and invasion of gastric cancer cells by activating Smad4 and inhibiting β-catenin signaling. Int J Oncol. 2012;41: 839–48. doi:10.3892/ijo.2012.1517

168. Du W, Wang S, Zhou Q, Li X, Chu J, Chang Z, et al. ADAMTS9 is a functional tumor suppressor through inhibiting AKT/mTOR pathway and associated with poor survival in gastric cancer. Oncogene. 2012; 1–10. doi:10.1038/onc.2012.359

169. Lee Y-C, Cheng T-H, Lee J-S, Chen J-H, Liao Y-C, Fong Y, et al. Nobiletin, a citrus flavonoid, suppresses invasion and migration involving FAK/PI3K/Akt and small GTPase signals in human gastric adenocarcinoma AGS cells. Mol Cell Biochem. 2011;347: 103–15. doi:10.1007/s11010-010-0618-z

170. Ho H-H, Chang C-S, Ho W-C, Liao S-Y, Lin W-L, Wang C-J. Gallic acid inhibits gastric cancer cells metastasis and invasive growth via increased expression of RhoB, downregulation of AKT/small GTPase signals and inhibition of NF-κB activity. Toxicol Appl Pharmacol. Elsevier Inc.; 2013;266: 76–85. doi:10.1016/j.taap.2012.10.019

171. Wang K, Liu R, Li J, Mao J, Lei Y, Wu J, et al. Quercetin induces protective autophagy in gastric cancer cells: involvement of Akt-mTOR- and hypoxia-induced factor 1α-mediated signaling. Autophagy. 2011;7: 966–78.

172. Lin H-H, Chen J-H, Chou F-P, Wang C-J. Protocatechuic acid inhibits cancer cell metastasis involving the down-regulation of Ras/Akt/NF-κB pathway and MMP-2 production by targeting RhoB activation. Br J Pharmacol. 2011;162: 237–54. doi:10.1111/j.1476-5381.2010.01022.x

173. Dar a a, Belkhiri a, El-Rifai W. The aurora kinase A regulates GSK-3beta in gastric cancer cells. Oncogene. 2009;28: 866–75. doi:10.1038/onc.2008.434

174. Snider JL, Allison C, Bellaire BH, Ferrero RL, Cardelli J a. The beta1 integrin activates JNK independent of CagA, and JNK activation is required for Helicobacter pylori CagA+-induced motility of gastric cancer cells. J Biol Chem. 2008;283: 13952–63. doi:10.1074/jbc.M800289200

175. Kim Y-M, Kim I-H, Nam T-J. Capsosiphon fulvescens glycoprotein inhibits AGS gastric cancer cell proliferation by downregulating Wnt-1 signaling. Int J Oncol. 2013;43: 1395–401. doi:10.3892/ijo.2013.2079

176. Hayakawa Y, Hirata Y, Nakagawa H, Sakamoto K, Hikiba Y, Kinoshita H, et al. Apoptosis signal-regulating kinase 1 and cyclin D1 compose a positive feedback loop contributing to tumor growth in gastric cancer. Proc Natl Acad Sci U S A. 2011;108: 780–5. doi:10.1073/pnas.1011418108

177. Kim S-M, Kim R, Ryu J-H, Jho E-H, Song K-J, Jang S-I, et al. Multinuclear giant cell formation is enhanced by down-regulation of Wnt signaling in gastric cancer cell line, AGS. Exp Cell Res. 2005;308: 18–28. doi:10.1016/j.yexcr.2005.04.002

178. Chun J, Joo EJ, Kang M, Kim YS. Platycodin D induces anoikis and caspase-mediated apoptosis via p38 MAPK in AGS human gastric cancer cells. J Cell Biochem. 2013;114: 456–70. doi:10.1002/jcb.24386

179. Lin H-H, Chen J-H, Huang C-C, Wang C-J. Apoptotic effect of 3,4-dihydroxybenzoic acid on human gastric carcinoma cells involving JNK/p38 MAPK signaling activation. Int J Cancer. 2007;120: 2306–16. doi:10.1002/ijc.22571

180. Ding Y, Li X-R, Yang K-Y, Huang L-H, Hu G, Gao K. Proteomics Analysis of Gastric Epithelial AGS Cells Infected with Epstein-Barr Virus. Asian Pac J Cancer Prev. 2013;14: 367–72.

181. Shin DY, Sung Kang H, Kim G-Y, Kim W-J, Yoo YH, Choi YH. Decitabine, a DNA methyltransferases inhibitor, induces cell cycle arrest at G2/M phase through p53-independent pathway in human cancer cells. Biomed Pharmacother. Elsevier Masson SAS; 2013;67: 305–311. doi:10.1016/j.biopha.2013.01.004

182. Höcker M, Rosenberg I, Xavier R, Henihan RJ, Wiedenmann B, Rosewicz S, et al. Oxidative stress activates the human histidine decarboxylase promoter in AGS gastric cancer cells. J Biol Chem. 1998;273: 23046–54.

183. Mishra P, Senthivinayagam S, Rangasamy V, Sondarva G, Rana B. Mixed lineage kinase-3/JNK1 axis promotes migration of human gastric cancer cells following gastrin stimulation. Mol Endocrinol. 2010;24: 598–607. doi:10.1210/me.2009-0387

184. Ho C-C, Lai K-C, Hsu S-C, Kuo C-L, Ma C-Y, Lin M-L, et al. Benzyl isothiocyanate (BITC) inhibits migration and invasion of human gastric cancer AGS cells via suppressing ERK signal pathways. Hum Exp Toxicol. 2011;30: 296–306. doi:10.1177/0960327110371991

185. Ancha HR, Kurella RR, Stewart C a, Damera G, Ceresa BP, Harty RF. Histamine stimulation of MMP-1(collagenase-1) secretion and gene expression in gastric epithelial cells: role of EGFR transactivation and the MAP kinase pathway. Int J Biochem Cell Biol. 2007;39: 2143–52. doi:10.1016/j.biocel.2007.06.003

186. Hotz B, Keilholz U, Fusi A, Buhr HJ, Hotz HG. In vitro and in vivo antitumor activity of cetuximab in human gastric cancer cell lines in relation to epidermal growth factor receptor (EGFR) expression and mutational phenotype. Gastric Cancer. 2012;15: 252–64. doi:10.1007/s10120-011-0102-9

187. Gong M, Meng L, Jiang B, Zhang J, Yang H, Wu J, et al. p37 from Mycoplasma hyorhinis promotes cancer cell invasiveness and metastasis through activation of MMP-2 and followed by phosphorylation of EGFR. Mol Cancer Ther. 2008;7: 530–7. doi:10.1158/1535-7163.MCT-07-2191

188. Caruso R, Pallone F, Fina D, Gioia V, Peluso I, Caprioli F, et al. Protease-activated receptor-2 activation in gastric cancer cells promotes epidermal growth factor receptor trans-activation and proliferation. Am J Pathol. 2006;169: 268–78. doi:10.2353/ajpath.2006.050841

189. Heindl S, Eggenstein E, Keller S, Kneissl J, Keller G, Mutze K, et al. Relevance of MET activation and genetic alterations of KRAS and E-cadherin for cetuximab sensitivity of gastric cancer cell lines. J Cancer Res Clin Oncol. 2012; 843–858. doi:10.1007/s00432-011-1128-4

190. Abdel-Latif MMM, Windle HJ, Fitzgerald KA, Ang YS, Eidhin DN, Li-Weber M, et al. Helicobacter pylori activates the early growth response 1 protein in gastric epithelial cells. Infect Immun. 2004;72: 3549–60. doi:10.1128/IAI.72.6.3549-3560.2004

191. Wang J, Gui Z, Deng L, Sun M, Guo R, Zhang W, et al. c-Met upregulates aquaporin 3 expression in human gastric carcinoma cells via the ERK signalling pathway. Cancer Lett. Elsevier Ireland Ltd; 2012;319: 109–17. doi:10.1016/j.canlet.2011.12.040

192. Wang Q, Liu X, Zhou J, Huang Y, Zhang S, Shen J, et al. Ribonucleotide reductase large subunit m1 predicts poor survival due to modulation of proliferative and invasive ability of gastric cancer. PLoS One. 2013;8: e70191. doi:10.1371/journal.pone.0070191

193. Ge Z, Zhu Y-L, Zhong X, Yu J-K, Zheng S. Discovering differential protein expression caused by CagA-induced ERK pathway activation in AGS cells using the SELDI-ProteinChip platform. World J Gastroenterol. 2008;14: 554–62.

194. Ding S-Z, Smith MF, Goldberg JB. Helicobacter pylori and mitogen-activated protein kinases regulate the cell cycle, proliferation and apoptosis in gastric epithelial cells. J Gastroenterol Hepatol. 2008;23: e67–78. doi:10.1111/j.1440-1746.2007.04912.x

195. Baek MK, Kim MH, Jang HJ, Park JS, Chung IJ, Shin BA, et al. EGF stimulates uPAR expression and cell invasiveness through ERK, AP-1, and NF-kappaB signaling in human gastric carcinoma cells. Oncol Rep. 2008;20: 1569–75. doi:10.3892/or

196. Jang SH, Cho S, Lee E-S, Kim JM, Kim H. The phenyl-thiophenyl propenone RK-I-123 reduces the levels of reactive oxygen species and suppresses the activation of NF-κB and AP-1 and IL-8 expression in Helicobacter pylori-infected gastric epithelial AGS cells. Inflamm Res. 2013; doi:10.1007/s00011-013-0621-4

197. Choi IJ, Kim JS, Kim JM, Jung HC, Song IS. Effect of inhibition of extracellular signal-regulated kinase 1 and 2 pathway on apoptosis and bcl-2 expression in Helicobacter pylori-infected AGS cells. Infect Immun. 2003;71: 830–7. doi:10.1128/IAI.71.2.830

198. Chen F-L, Wang X-Z, Li J-Y, Yu J-P, Huang C-Y, Chen Z-X. 12-lipoxygenase induces apoptosis of human gastric cancer AGS cells via the ERK1/2 signal pathway. Dig Dis Sci. 2008;53: 181–7. doi:10.1007/s10620-007-9841-1

199. Park SJ, Kim YY, Lim JY, Seo GJ, Kim J, Park SI, et al. Opposite role of Ras in tumor necrosis factor-alpha-induced cell cycle regulation: competition for Raf kinase. Biochem Biophys Res Commun. 2001;287: 1140–7. doi:10.1006/bbrc.2001.5713

200. Brandt S, Wessler S, Hartig R, Backert S. Helicobacter pylori activates protein kinase C delta to control Raf in MAP kinase signalling: role in AGS epithelial cell scattering and elongation. Cell Motil Cytoskeleton. 2009;66: 874–92. doi:10.1002/cm.20373

201. Jang SH, Lim JW, Kim H. Beta-carotene inhibits Helicobacter pylori-induced expression of inducible nitric oxide synthase and cyclooxygenase-2 in human gastric epithelial AGS cells. J Physiol Pharmacol. 2009;60 Suppl 7: 131–7.

202. Steigedal TS, Prestvik WS, Selvik L-KM, Fjeldbo CS, Bruland T, Lægreid A, et al. Gastrin-induced proliferation involves MEK partner 1 (MP1). In Vitro Cell Dev Biol Anim. 2013;49: 162–9. doi:10.1007/s11626-013-9588-2

203. Yang M-D, Lai K-C, Lai T-Y, Hsu S-C, Kuo C-L, Yu C-S, et al. Phenethyl isothiocyanate inhibits migration and invasion of human gastric cancer AGS cells through suppressing MAPK and NF-kappaB signal pathways. Anticancer Res. 2010;30: 2135–43.

204. Zhou J, Xie Y, Zhao Y, Wang S, Li Y. Human gastrin mRNA expression up-regulated by Helicobacter pylori CagA through MEK/ERK and JAK2-signaling pathways in gastric cancer cells. Gastric Cancer. 2011;14: 322–31. doi:10.1007/s10120-011-0044-2

205. Huang Y, Zhu Z, Sun M, Wang J, Guo R, Shen L, et al. Critical role of aquaporin-3 in the human epidermal growth factor-induced migration and proliferation in the human gastric adenocarcinoma cells. Cancer Biol Ther. 2010;9: 1000–7. doi:10.4161/cbt.9.12.11705

206. Kang W, Nielsen O, Fenger C, Leslie G, Holmskov U, Reid KBM. Induction of DMBT1 expression by reduced ERK activity during a gastric mucosa differentiation-like process and its association with human gastric cancer. Carcinogenesis. 2005;26: 1129–37. doi:10.1093/carcin/bgi045

207. Higashi H, Nakaya A, Tsutsumi R, Yokoyama K, Fujii Y, Ishikawa S, et al. Helicobacter pylori CagA induces Ras-independent morphogenetic response through SHP-2 recruitment and activation. J Biol Chem. 2004;279: 17205–16. doi:10.1074/jbc.M309964200

208. Yamamura Y, Lee WL, Inoue K, Ida H, Ito Y. RUNX3 cooperates with FoxO3a to induce apoptosis in gastric cancer cells. J Biol Chem. 2006;281: 5267–76. doi:10.1074/jbc.M512151200

209. Kim N, Kim CH, Ahn D-W, Lee KS, Cho S-J, Park JH, et al. Anti-gastric cancer effects of celecoxib, a selective COX-2 inhibitor, through inhibition of Akt signaling. J Gastroenterol Hepatol. 2009;24: 480–7. doi:10.1111/j.1440-1746.2008.05599.x

210. Hirata Y, Maeda S, Ohmae T, Shibata W, Yanai A, Ogura K, et al. Helicobacter pylori induces IkappaB kinase alpha nuclear translocation and chemokine production in gastric epithelial cells. Infect Immun. 2006;74: 1452–61. doi:10.1128/IAI.74.3.1452-1461.2006

211. Rieke C, Papendieck A, Sokolova O, Naumann M. Helicobacter pylori-induced tyrosine phosphorylation of IKKβ contributes to NF-κB activation. Biol Chem. 2011;392: 387–93. doi:10.1515/BC.2011.029

212. Kwon M-J, Nam T-J. A polysaccharide of the marine alga Capsosiphon fulvescens induces apoptosis in AGS gastric cancer cells via an IGF-IR-mediated PI3K/Akt pathway. Cell Biol Int. 2007;31: 768–75. doi:10.1016/j.cellbi.2007.01.010

213. Zhang Y, Shi Y, Li X, Du W, Luo G, Gou Y, et al. Inhibition of the p53-MDM2 interaction by adenovirus delivery of ribosomal protein L23 stabilizes p53 and induces cell cycle arrest and apoptosis in gastric cancer. J Gene Med. 2010;12: 147–56. doi:10.1002/jgm.1424

214. Yin Y, Grabowska AM, Clarke P a, Whelband E, Robinson K, Argent RH, et al. Helicobacter pylori potentiates epithelial:mesenchymal transition in gastric cancer: links to soluble HB-EGF, gastrin and matrix metalloproteinase-7. Gut. 2010;59: 1037–45. doi:10.1136/gut.2009.199794

215. Wroblewski LE, Noble P-JM, Pagliocca A, Pritchard DM, Hart CA, Campbell F, et al. Stimulation of MMP-7 (matrilysin) by Helicobacter pylori in human gastric epithelial cells: role in epithelial cell migration. J Cell Sci. 2003;116: 3017–26. doi:10.1242/jcs.00518

216. Datta De D, Datta A, Bhattacharjya S, Roychoudhury S. NF-kappaB mediated transcriptional repression of Acid modifying hormone gastrin. PLoS One. 2013;8: e73409. doi:10.1371/journal.pone.0073409

217. Yang Z, Yuan X-G, Chen J, Luo S-W, Luo Z-J, Lu N-H. Reduced expression of PTEN and increased PTEN phosphorylation at residue Ser380 in gastric cancer tissues: a novel mechanism of PTEN inactivation. Clin Res Hepatol Gastroenterol. Elsevier Masson SAS; 2013;37: 72–9. doi:10.1016/j.clinre.2012.03.002

218. Xue Y, Bi F, Zhang X, Pan Y, Liu N, Zheng Y, et al. Inhibition of endothelial cell proliferation by targeting Rac1 GTPase with small interference RNA in tumor cells. Biochem Biophys Res Commun. 2004;320: 1309–1315. doi:10.1016/j.bbrc.2004.06.088

219. Kwon H-K, Bae G-U, Yoon J-W, Kim YK, Lee H-Y, Lee H-W, et al. Constitutive activation of p70S6k in cancer cells. Arch Pharm Res. 2002;25: 685–90.

220. Yu EJ, Lee Y, Rha SY, Kim TS, Chung HC, Oh BK, et al. Angiogenic factor thymidine phosphorylase increases cancer cell invasion activity in patients with gastric adenocarcinoma. Mol Cancer Res. 2008;6: 1554–66. doi:10.1158/1541-7786.MCR-08-0166

221. He J, Sheng T, Stelter A a, Li C, Zhang X, Sinha M, et al. Suppressing Wnt signaling by the hedgehog pathway through sFRP-1. J Biol Chem. 2006;281: 35598–602. doi:10.1074/jbc.C600200200

222. Pan H-C, Lai D-W, Lan K-H, Shen C-C, Wu S-M, Chiu C-S, et al. Honokiol thwarts gastric tumor growth and peritoneal dissemination by inhibiting Tpl2 in an orthotopic model. Carcinogenesis. 2013;00: 1–12. doi:10.1093/carcin/bgt243

223. Zheng Q, Zhao L-Y, Kong Y, Nan K-J, Yao Y, Liao Z-J. CDK-associated Cullin 1 can promote cell proliferation and inhibit cisplatin-induced apoptosis in the AGS gastric cancer cell line. World J Surg Oncol. World Journal of Surgical Oncology; 2013;11: 5. doi:10.1186/1477-7819-11-5

224. Li X, Fan R, Zou X, Hong L, Gao L, Jin H, et al. [Reversal of multidrug resistance of gastric cancer cells by down-regulation of CIAPIN1 with CIAPIN1 siRNA]. Mol Biol (Mosk). 2006;42: 102–9.

225. Li R, Chen W-C, Pang X-Q, Tian W-Y, Wang W-P, Zhang XG. Effect of PI3K gene silencing on growth, migration and related proteins expression of CD40 signal-mediated gastric cancer cells. Mol Biol Rep. 2013;40: 999–1008. doi:10.1007/s11033-012-2141-7
