# Supplementary material for: Discovery of Drug Synergies in Gastric Cancer Cells Predicted by Logical Modeling
Source: PLoS Comput Biol. 2015 Aug 28;11(8):e1004426. doi: 10.1371/journal.pcbi.1004426 (PMC4567168; doi:10.1371/journal.pcbi.1004426)
Supplement: S8 Table — (DOC) [file pcbi.1004426.s012.doc]

|  | **Node** | **Target value** | **Logical rule** |
| --- | --- | --- | --- |
| **Inputs** | betacatenin | {0, 1} | input |
|  | GSK3 | {0, 1} | input |
| **Internal nodes** | AKT | 1 | [PI3K & (betacatenin | GSK3 | !TAK1)] | [!ERK & !TAK1 & !AKT & GSK3] |
|  | ERK | 1 | MEK \| (!ERK & !AKT) |
|  | MEK | 1 | !(ERK & TAK1 & p38alpha & AKT & !GSK3 & !betacatenin) |
|  | p38alpha | 1 | !ERK & !p38alpha |
|  | PI3K | 1 | !(TAK1 & ERK) \| betacatenin \| GSK3 |
|  | TAK1 | 1 | !p38alpha |
| **Ouput nodes** | Antisurvival | 3 | !ERK & !TAK1 & !AKT & p38alpha & PI3K |
|  |  | 2 | !AKT & !ERK & TAK1 & p38alpha & PI3K & (GSK3 \| betacatenin) \| [!AKT & !TAK1 & !(p38alpha & PI3K & !ERK)] |
|  |  | 1 | [!ERK & !TAK1 & AKT & !p38alpha] \| [TAK1 & ERK & !AKT] \| [TAK1 & AKT & !ERK & !p38alpha] \| [!ERK & TAK1 & !AKT & !(p38alpha & PI3K & (GSK3 \| betacatenin))] |
|  | Prosurvival | 3 | ERK & PI3K & (!TAK1 \| betacatenin) |
|  |  | 2 | ((TAK1 & betacatenin) \| !TAK1 ) & !(ERK & PI3K)] |
|  |  | 1 | ERK & PI3K & GSK3 & TAK1 & !betacatenin |

Supplementary Table S8: **Logical formulae associated with the ten components of the reduced AGS reduced logical model.** The symbols “!”, “&” and “|” (used in GINsim) stand for the classical Boolean operators NOT, AND and OR. These operators have decreasing priorities in that orders, and parentheses are used at some places to overwrite this order when needed. Only the formula for non zero target values are specified, as GINsim assign a zero value per default to all non specified interaction combinations. All components but Antisurvival and Prosurvival are modelled by Boolean variables. The output components Antisurvival and Prosurvival can take four different values, 0, 1, 2 and 3 (as in the complete models). Their relatively complicate rules result from the higher number of direct regulators of these nodes following reduction. In several cases, the logical formula has been organized in different terms (between square brackets) on successive rows, connected with OR operators. Note that betacatenin and GSK3 correspond to input (unregulated) components as a result of the reduction process.
